# Supplementary material for: Multimethods study comparing the experiences of medical clinical academics with nurses, midwives and allied health professionals pursuing a clinical academic career
Source: BMJ Open. 2021 Apr 1;11(4):e043270. doi: 10.1136/bmjopen-2020-043270 (PMC8023736; doi:10.1136/bmjopen-2020-043270)
Supplement: Supplementary data [file bmjopen-2020-043270supp003.pdf]

Clinical Academic Survey for medics

Page 1: Clinical academic survey

Health Education England (HEE) PG Specialty School of Clinical Academic Training in the East Midlands

We are sending you this survey because we are interested in finding out more about your experiences of pursuing a clinical academic pathway. A similar survey has already been completed by nurses, midwives and allied health professionals in the East Midlands. The aim of this further study is to compare clinical academic experiences of different professions.

Completing the survey will take approximately 10 minutes and as a thank you for your time there is a chance to **win a £50 Amazon voucher**.

Please be assured that all of your responses will be treated confidentially. Also, any quotes from the data that we may use when disseminating the results, will be anonymised.

All returned responses will be entered into a **prize draw** for a chance to **win a £50 Amazon voucher!** Please return the form by midnight on **15th July, 2019** and provide your name and email address here so that we can contact the prize winner.

Thank you very much in advance for your help. Good luck in the prize draw!

Trusson D, *et al. BMJ Open* 2021; 11:e043270. doi: 10.1136/bmjopen-2020-043270

Page 2: Part 1 - About you

Which age group are you in? *Optional*

- ○ 20-30
- ○ 31-40
- ○ 41-50
- ○ 51+

How would you describe your gender?

- ○ Male
- ○ Female
- ○ Other
- ○ Prefer not to say

How would you describe your ethnicity? (optional)

Current employment

- ☐ NHS
- ☐ University

Who are you employed by?

What is the name of the clinical practice where you are employed?

Which postgraduate specialty school are you a member of?

- Foundation School
- School of Anaesthesia
- School of Emergency Medicine
- Primary Care
- School of Medicine
- Obstetrics and Gynaecology
- Paediatrics
- Pathology
- Psychiatry
- Public Health
- Radiology
- Surgery

Please tell us the name of your clinical training programme

Trusson D, *et al. BMJ Open* 2021; 11:e043270. doi: 10.1136/bmjopen-2020-043270

Page 3: Do you have a PhD/MD or are you studying for one?

If you are studying for a PhD, which University is your PhD affiliated with?

- ☐ University of Leicester
- ☐ University of Nottingham
- ☐ Other

Please give the name of University that your PhD is affiliated with

For current PhD/MD students: When did you start your PhD/MD? (month/year)

Who is funding your PhD?

Please give details of your PhD funding

Where are you on the PhD/MD journey?

- ☐ Year 1

Trusson D, *et al. BMJ Open* 2021; 11:e043270. doi: 10.1136/bmjopen-2020-043270

- ☐ Year 2
- ☐ Year 3
- ☐ Year 4
- ☐ Year 5
- ☐ Year 6
- ☐ Submitted but not awarded

What is your anticipated submission date? (month/year)

Page 4

Please give brief details below of what you have done as part of your academic training:

Please describe ways in which your academic training has improved your skills as a clinician

Have you presented your research:

- ☐ Locally
- ☐ Nationally
- ☐ Internationally
- ☐ All the above

Please provide details of how you have disseminated your research (e.g. publications, conference presentations/posters). If possible, please provide links or cut and paste details.

Have you had any successful grant applications?

- Yes
- No

If so, please provide details (e.g. the funding body, research project, grant reference (if any), and whether you were the lead or co-applicant)

Page 5: Part 2. Becoming a clinical academic

In this part of the questionnaire, we want to find out what the term ‘clinical academic’ means to you, and how the clinical academic training you have received has influenced your career.

The description ‘clinical academic’ is varied. We want to try to find a way to best explain what the role is. Please explain what you understand by the title ‘clinical academic’:

Do you consider yourself to be a clinical academic?

- Yes
- No

If so why? (for example, do you have funded research and clinical sessions?)

How has your academic training helped you in your career? (if applicable)

Trusson D, *et al. BMJ Open* 2021; 11:e043270. doi: 10.1136/bmjopen-2020-043270

If applicable, how did you overcome any challenges in achieving success and/or progressing to the next level in your clinical academic career?

What types of support did you find most helpful during your clinical academic training?

Do you anticipate any challenges in pursuing a clinical academic career? For example if you want to take breaks for parental leave or work part time in the future?

Have your goals changed during your training?

- Yes
- No

If so, please explain how and why your goals have changed:

Trusson D, *et al. BMJ Open* 2021; 11:e043270. doi: 10.1136/bmjopen-2020-043270

Trusson D, *et al. BMJ Open* 2021; 11:e043270. doi: 10.1136/bmjopen-2020-043270

Page 6

Are there any resources that you found beneficial in developing your clinical academic role which you would be happy to share for the benefit of others?

These might include, for example, websites where you have found useful advice and information.

If you have any resources that you think might be useful, please send them as an attachment to the following email: diane.trusson@nottingham.ac.uk . Alternatively, please give link(s) to electronic resources here:

What advice would you give to people who are considering a clinical academic career?

Is there anything else that you want to add?

Page 7: Part 3. Would you be willing to talk about your experiences?

Would you be interested in taking part in a focus group to discuss your experiences?

- Yes
- No

If so, please provide your contact details here:

We would also like to develop some case studies to demonstrate the impact that undertaking a clinical academic pathway can have both in relation to your career and your clinical practice. Would you be willing to be interviewed about your experiences?

- Yes
- No

If so, please provide your contact details here:

Trusson D, *et al. BMJ Open* 2021; 11:e043270. doi: 10.1136/bmjopen-2020-043270

Page 8: Thank you for taking the time to complete this survey, your responses are very important to us.

Key for selection options

6.a - Who are you employed by?

University of Leicester
University of Nottingham
University Hospitals of Leicester
Nottingham University Hospitals
Partnership Trust
Other

8.a - Who is funding your PhD?

NIHR
Wellcome Trust
Medical Research Council (MRC)
Other

Trusson D, *et al. BMJ Open* 2021; 11:e043270. doi: 10.1136/bmjopen-2020-043270
