# Supplementary material for: Multimethods study comparing the experiences of medical clinical academics with nurses, midwives and allied health professionals pursuing a clinical academic career
Source: BMJ Open. 2021 Apr 1;11(4):e043270. doi: 10.1136/bmjopen-2020-043270 (PMC8023736; doi:10.1136/bmjopen-2020-043270)
Supplement: Author's manuscript [file bmjopen-2020-043270.draft_revisions.pdf]

BMJ Open is committed to open peer review. As part of this commitment we make the peer review history of every article we publish publicly available.

When an article is published we post the peer reviewers' comments and the authors' responses online. We also post the versions of the paper that were used during peer review. These are the versions that the peer review comments apply to.

The versions of the paper that follow are the versions that were submitted during the peer review process. They are not the versions of record or the final published versions. They should not be cited or distributed as the published version of this manuscript.

BMJ Open is an open access journal and the full, final, typeset and author-corrected version of record of the manuscript is available on our site with no access controls, subscription charges or pay-per-view fees (http://bmjopen.bmj.com).

If you have any questions on BMJ Open's open peer review process please email info.bmjopen@bmj.com

BMJ Open

A mixed-methods study comparing the experiences of medical clinical academics with nurses, midwives and allied health professionals pursuing a clinical academic career.

Journal:	*BMJ Open*
Manuscript ID	bmjopen-2020-043270
Article Type:	Original research
Date Submitted by the Author:	29-Jul-2020
Complete List of Authors:	Trusson, Diane; University of Nottingham, School of Medicine<br>Rowley, Emma; University of Nottingham, School of Medicine. National Institute for Health Research (NIHR) Applied Research Collaboration East Midlands (ARC EM)<br>Barratt, Jonathan; University of Leicester College of Medicine Biological Sciences and Psychology
Keywords:	EDUCATION & TRAINING (see Medical Education & Training), MEDICAL EDUCATION & TRAINING, QUALITATIVE RESEARCH

SCHOLARONE™
Manuscripts

I, the Submitting Author has the right to grant and does grant on behalf of all authors of the Work (as defined in the below author licence), an exclusive licence and/or a non-exclusive licence for contributions from authors who are: i) UK Crown employees; ii) where BMJ has agreed a CC-BY licence shall apply, and/or iii) in accordance with the terms applicable for US Federal Government officers or employees acting as part of their official duties; on a worldwide, perpetual, irrevocable, royalty-free basis to BMJ Publishing Group Ltd ("BMJ") its licensees and where the relevant Journal is co-owned by BMJ to the co-owners of the Journal, to publish the Work in this journal and any other BMJ products and to exploit all rights, as set out in our licence.

The Submitting Author accepts and understands that any supply made under these terms is made by BMJ to the Submitting Author unless you are acting as an employee on behalf of your employer or a postgraduate student of an affiliated institution which is paying any applicable article publishing charge ("APC") for Open Access articles. Where the Submitting Author wishes to make the Work available on an Open Access basis (and intends to pay the relevant APC), the terms of reuse of such Open Access shall be governed by a Creative Commons licence – details of these licences and which Creative Commons licence will apply to this Work are set out in our licence referred to above.

Other than as permitted in any relevant BMJ Author's Self Archiving Policies, I confirm this Work has not been accepted for publication elsewhere, is not being considered for publication elsewhere and does not duplicate material already published. I confirm all authors consent to publication of this Work and authorise the granting of this licence.

For peer review only

A mixed-methods study comparing the experiences of medical clinical academics with nurses, midwives and allied health professionals pursuing a clinical academic career.

Author names

Diane Trusson, Emma Rowley and Jonathan Barratt

Correspondence address: Dr Diane Trusson, NIHR Applied Research Collaboration for East Midlands (ARC EM), Institute of Mental Health, University of Nottingham Innovation Park, Triumph Road, Nottingham, NG7 2TU. Email: Diane.Trusson@nottingham.ac.uk ;Phone: 0115 7484249

Diane Trusson PhD: NIHR Applied Research Collaboration for East Midlands (ARC EM); University of Nottingham School of Medicine, UK. Orcid ID: 0000-0002-6995-1192

Emma Rowley PhD: NIHR Applied Research Collaboration for East Midlands (ARC EM); Nottingham University Business School, Nottingham, UK. Orcid: 0000-0002-6668-1350

Jonathan Barratt PhD FRCP: University of Leicester and Leicester General Hospital, Leicester, UK. Orcid ID: 0000-0002-9063-7229

Key words: Clinical Academic careers; Medical Clinical Academics; Nurses, midwives and AHPs; Mixed methods; Interviews

Word count: 3994

For peer review only - http://bmjopen.bmj.com/site/about/guidelines.xhtml

ABSTRACT:

Objectives: This study aimed to compare experiences of medical clinical academics (MCAs) with those of nurses, midwives and AHPs (NMAHPs) pursuing a clinical academic career.

Design: Both sets of participants completed similar online surveys followed by in-depth interviews to explore emerging themes.

Setting: The research was conducted in the East Midlands of England, encompassing two HEIs and four NHS Trusts.

Participants: Surveys were completed by 67 NMAHPs and 73 MCA trainees. Sixteen participants from each group were interviewed following a similar interview schedule.

Results: The survey data revealed notable differences in demographics of the two study populations, reflecting their different career structures. MCAs were younger and they all combined clinical and academic training, lengthening the time before qualification. In contrast, most NMAHPs had been in their clinical post for some years before embarking on a clinical academic pathway. Both routes had financial and personal repercussions and participants faced similar obstacles. However, there was also evidence of wide-ranging benefits from combining clinical and academic roles.

Conclusions: Variations in experiences between the two study populations highlight a need for a clear academic pathway for all health professionals,

For peer review only

as well as sufficient opportunities post-PhD to enable clinical academics to fully utilise their dual skills.

STRENGTHS AND LIMITATIONS:

- First study to compare experiences of medical clinical academics with those of nurses, midwives and AHPs pursuing a clinical academic career.
- Mixed methods enabled both breadth and depth of data to be gathered for comparison purposes.
- Limited to one geographical area.

INTRODUCTION

Recognising the value of supporting clinicians to do research in their area of expertise, the National Institute for Health Research (NIHR) provides a range of schemes to encourage aspiring clinical academics.[1] For medical clinical academics (MCAs), the academic career path can begin at medical school with an intercalated degree or MB PhD programme, alongside other opportunities to be involved in research. Academic foundation programme (AFP) posts funded by Health Education England (HEE), offer protected academic time during foundation year 2. The NIHR Integrated Academic Training Pathway (IATP) supports academic clinical fellowships (ACFs) enabling academic training alongside specialty training with the aim of supporting entry onto a PhD programme, doctoral research fellowships and Clinical Lectureships (CLs) enabling postdoctoral clinicians

For peer review only - http://bmjopen.bmj.com/site/about/guidelines.xhtml

to split their time equally between clinical and academic training.[2] (See figure 1).

[Insert figure 1 here]

In parallel, the HEE and NIHR Integrated Clinical Academic (ICA) Programme provides research training awards for healthcare professionals (excluding doctors and dentists) wanting to develop careers combining clinical research and research leadership with continued clinical practice and professional development. These include internships, clinical academic fellowships, doctoral research fellowships, clinical and senior clinical lectureships, alongside bridging schemes.[3] In contrast to MCAs, the NMAHP clinical academic pathway awards can only be pursued post-graduation and upon completion of clinical preceptorship.

For both professional groups, benefits of pursuing a clinical academic career include intellectual stimulation and improved career prospects[4,5] as well as wide-ranging benefits for patient care.[6] Studies also identify challenges, including unsupportive work environments, competing pressures, scarcity of funding and senior academic appointments and difficulty achieving a good work/life balance.[7,8] Previous studies have focussed either on MCAs,[9,10] or on NMAHPs.[11] However, it remains unclear how the benefits and challenges associated with the clinical academic pathway compare between MCAs and NMAHPs. The current study addresses this gap.

For peer review only - http://bmjopen.bmj.com/site/about/guidelines.xhtml

In addition, concerns about a 'decline in the capacity of NHS staff to undertake, or even engage with research'[6,p6] as well as rates of attrition along the clinical academic career trajectory, particularly of women,[6] have made it increasingly important to identify factors that encourage and enable continuation to higher levels of clinical academia.[7]

The following section describes the design of two studies conducted in the East Midlands; one explored NMAHPs' clinical academic experiences, the other was a comparative study of MCAs. Of interest were similarities and differences between the two study populations in terms of demographics, motivation, and experiences of pursuing a clinical academic career.

METHODS

This manuscript has been prepared according to the Standards for Reporting Qualitative Research[12](see online supplementary file 1).

Study design

A Bristol Online Survey[13] was created for each study (see online supplementary files 2-3) to gather data from a relatively large number of respondents at different stages along the clinical academic pathway. This was followed by semi-structured interviews to enable a deeper exploration of individual experiences which can be lost in focus group interviews for example.[10]

Both the survey and interview guide were broadly similar for each sample (see online supplementary files 4-5). However, the MCA survey was modified to better reflect the medical IAT pathway, for example asking for

For peer review only - http://bmjopen.bmj.com/site/about/guidelines.xhtml

participants' medical specialty rather than job title. Furthermore, the MCA survey asked about anticipating challenges, such as taking parental leave or working part-time in the future and, in interviews, MCAs were asked for their perspectives on combining clinical academic careers with family life. These questions were prompted by concerns around diversity, particularly at higher levels,[6] and previous research suggesting that difficulty in achieving a good work-life balance could be a barrier to clinical academic career progression.[7] Although this question was not explicitly posed in the NMAHPs study, there were sufficient unsolicited data on this topic to allow a comparison of their respective experiences and giving credence for this topic meriting further investigation.

Recruitment

Participants were recruited from two HEIs and four large NHS Trusts in the East Midlands. A link to the survey was sent to a clinical academic network for NMAHPs[14] and further disseminated via Twitter. MCAs were accessed via email sent to a database of 263 MCA trainees. Both surveys were open for approximately 2 months. Potential participants had the opportunity to enter a prize draw as an incentive to participate.

Half of each survey's respondents indicated that their willingness to be interviewed. Quota sampling was used[15] to select 16 respondents from each study population, representing a range of demographics and stages of training. See tables 1 and 2 for interview sample characteristics:

Case study	Clinical role	Stage of study (at interview)	Age group	Gender

For peer review only - http://bmjopen.bmj.com/site/about/guidelines.xhtml

1	Nurse/midwife	Thesis pending	20-30	Female
2	Nurse/midwife	Year 2 PhD	41-50	Female
3	Nurse/midwife	Year 4 PhD	41-50	Female
4	Nurse/midwife	Post-doc	41-50	Female
5	Nurse/midwife	Year 3 PhD	41-50	Female
6	AHP	Year 4 PhD	41-50	Female
7	AHP	Year 2 PhD	41-50	Female
8	Nurse/midwife	Year 5 PhD	41-50	Female
9	Nurse/midwife	Post-doc	31-40	Male
10	AHP	Year 2 PhD	41-50	Female
11	AHP	Year 1 PhD	41-50	Female
12	AHP	Year 4 PhD	51+	Male
13	AHP	Year 3 PhD	31-40	Male
14	AHP	Year 3 PhD	41-50	Male
15	AHP	Post-doc	51+	Male
16	AHP	Year 1 PhD	31-40	Male

Table 1 NMAHP Interview sample characteristics

Case study (MCA)	Clinical training programme (at interview)	Age group	Gender
1	ACF	31-40	Female
2	Clinical lecturer	31-40	Female
3	Year 3 PhD	20-30	Female
4	ACF	20-30	Male
5	AFP	20-30	Male
6	AFP	20-30	Male
7	Year 2 PhD	31-40	Male
8	ACF	31-40	Female
9	Year 4 PhD	31-40	Male
10	Clinical lecturer	31-40	Female
11	ACF	20-30	Male
12	Clinical lecturer	31-40	Female
13	Clinical lecturer	31-40	Female
14	Clinical lecturer	31-40	Male
15	Year 2 PhD	31-40	Female
16	ACF	31-40	Female

Table 2 MCA Interview sample characteristics

For peer review only

Data collection

Acknowledging clinical academics' limited availability, individual interviews were conducted at a time and place to suit the interviewee. This was usually their place of work or a public place (e.g. café). All interviews began by asking about their experiences of being a clinical academic, enabling the participant to prioritise what they considered to be the most

For peer review only - http://bmjopen.bmj.com/site/about/guidelines.xhtml

salient aspects of their experiences. Although the interviews were flexible, a pre-prepared list of questions enabled responses to be compared within the sample and also between research populations.[16]

All interviews were digitally recorded with the participants' permission.

Research Team

The team consisted of a medical sociologist, a capacity development manager and a clinical academic from the respective study population who helped to tailor the survey and interview questions and facilitate participant recruitment. The first author who conducted all the interviews, was an experienced social researcher with no prior relationship with the participants. All authors contributed to the study design, data analysis and preparing the article.

Ethical considerations

Following Health Research Authority[17] guidance, ethical approval was not required for this study because participants were recruited by virtue of their participation in educational programmes, not their NHS status. Nevertheless, good research governance was observed, i.e. any identifying information given in order to enter the prize draw and/or offer to be interviewed, was removed prior to analysis. Interviewees were informed about the purpose of the research, their right to withdraw from the study and how their (anonymised) data would be used. In addition, participants were assured that data would be stored confidentially on

For peer review only

secure University systems. Verbal consent was obtained prior to each interview.

Data processing

Data from the surveys were collated and numerical data were summarized in graph form using BOS software.[13] Interviews were transcribed professionally and then all identifying data were removed prior to analysis. To ensure anonymity, NMAHP survey respondents are identified as SR1-SR67 or case study number CS1-CS16, plus their role. MCA survey respondents are identified by MSR1-MSR73, or case study number (MCA1-MCA16) with their stage of training. In addition, data extracts used in the discussion were sent to the relevant participants to confirm that they could not be identified by the contents.

Analysis:

For this article, survey and interview data from both studies were combined. Themes emerged from the data through an inductive process involving multiple readings of the data.[18]This reflexive process is used as an iterative way of developing insight.[19]

The themes selected for discussion in this article are those most relevant to comparing the clinical academic experiences of NMAHPs and MCAs.

Rigour

Combining survey and interview data increased the credibility of the findings.[20] During the analysis process, all authors discussed and agreed the emerging themes to ensure reliability.[18]

For peer review only - http://bmjopen.bmj.com/site/about/guidelines.xhtml

Patient and public involvement

No patients were involved.

RESULTS

The surveys were completed by 67 NMAHPs and 73 MCAs who described their job role (figure 2) or stage of training (figure 3) as follows:

[Insert figures 2 and 3 here]

In the following section, similarities and differences will be discussed in terms of demographics, motivation, clinical academic training experiences and career pathways. There is insufficient room here to discuss the many rich data gathered on the topic of combining a clinical academic career and family life, which will be discussed in a forthcoming paper.

Gender:

[Insert figure 4 here]

Figure 4 reveals relatively equal numbers of male (41) and female (32) MCAs survey respondents, whereas female NMAHPs outnumbered their male counterparts by 5:1. These figures are representative of the gender divide for these occupations nationally.[21] However, it is worth noting that these data were gathered from MCAs at an early stage in their careers and 'although approximately 50% of pre-doctoral ACFs are female…the percentage of female fellows declines with increasing seniority of award'.[1.p1]

For peer review only - http://bmjopen.bmj.com/site/about/guidelines.xhtml

Age:

There were major differences between the two populations in terms of age of the survey respondents:

[Insert figure 5 here]

Figure 5 reveals substantially higher numbers of MCAs in the 20-30 age group compared with NMAHPs. This reflects the career structures of the two populations. With medical trainees being encouraged to undertake academic training in parallel with their clinical training, they would be more likely to fall into the 20-30 bracket.

Although the number of NMAHPs aged 51+ was not high, it contrasted with the lack of any MCAs in this age group. This is consistent with findings which revealed that NMAHPs tend to commence the clinical academic pathway after establishing their careers whereas MCAs practising regionally who are 50+ are no longer on the training pathway; hence they are not captured in this dataset.

Embarking on a clinical academic career

In contrast to medical trainees who are encouraged to embark on the clinical academic pathway early in their career, academic training was a step-change in NMAHPs' career trajectory. This seemed to lead to a difference in attitudes towards participants in the two studies. For example, younger NMAHPs had to overcome certain prejudices:

For peer review only - http://bmjopen.bmj.com/site/about/guidelines.xhtml

> We've got this traditional sort of snobbery that you have to serve X amount of years before you are worthy to do academia (CS8Midwife).

This reveals how NMAHPs might be discouraged from pursuing a clinical academic career at an early stage, potentially holding talent back.[11]

Motivation

Both sets of participants described being motivated to do research by a drive to improve patient care:

> What got my brain ticking was why one patient presented a certain way, and not another patient with exactly the same disease. I always wanted to know the why (MCA2CL).

However, findings revealed the range of research made possible by approaching it from different perspectives. For example:

> We don't want to just hand [research] over to medics. Because they see the women who are ill or need help, but we also need to improve care for the women who go through a normal pathway (CS4Midwife).

This is supported by the NIHR:

> We need to back away from the doctor-driven research narrative…[nurses, midwives and AHPs] are the experts in what they do – they must be supported to do research because they are

For peer review only

> the ones who are seeing the gaps, and who know the questions that must be answered.[22,p3]

Another motivating factor for many MCAs was their early experiences of research:

> When I was a medical student [I took] one year out to do full-time research and I loved it (MCA9PhD).

This indicates the benefit of encouraging research, starting at undergraduate level.[6] In contrast, NMAHPs' prior exposure to research training varied according to their profession. For example, research has been part of the curriculum for student physiotherapists for many years, whereas nurses have only comparatively recently been required to undertake degree-level training.[23] This may explain why AHPs outnumbered nurses in the NMAHP study although changes may occur as more nurses emerge from training having had research experience.

However, while NMAHPs described choosing their research topic, the data revealed inconsistencies in the opportunities for MCAs to pursue their own personal research interests. One participant described the process for assigning research topics:

> We all put our names in a hat...I ended up with my third or fourth choice (MCA3ACF).

Although this practice was only reported by participants from one training provider, it had the potential to deter people from doing research. For

For peer review only - http://bmjopen.bmj.com/site/about/guidelines.xhtml

example, one academic clinical fellow described being assigned a project which was not achievable in the 9 months available. Consequently, she had decided to focus solely on clinical training while her family were young.

> I haven't regretted doing it but…I haven't enjoyed it enough to keep going with it while I'm bringing up a family (MCA1ACF).

Similarly, several female NMAHPs described waiting until their children reached school age before embarking on a clinical academic pathway:

> My youngest daughter started school, so I had a bit of head space to think about where I wanted to take my career (CS2Nurse).

Comparisons with MCAs

NMAHPs expressed frustration at what they perceived to be an 'easier' route for MCAs:

> For them it's a perfectly normal part of your career to do a PhD...it's such a shame that the culture is slow to be absorbed into the nursing profession (CS5Nurse).

> A doctor I've been working with was doing an MD…He said it was more prestigious to do a PhD, so they've just swapped him. Do you know the pains that I went through to get PhD funding and you've just swapped? (CS11AHP).

For peer review only - http://bmjopen.bmj.com/site/about/guidelines.xhtml

This participant seemed particularly unhappy by what was perceived as unfair financial penalisation:

> [Doctors] are given 2-3 days a week to do research, plus travel and parking expenses. My department doesn't have a training budget anymore. Any extra training you get, you pay out of your own pocket; you don't get study leave, you have to take annual leave. It's not equitable is it?' (CS11AHP).

The benefits of this support were confirmed by MCAs:

> I've been to lots of conferences…I've met a lot of influential people [its] really beneficial, for creating opportunities for my future (MCA3PhD)

Although the medical clinical academic route was better designed and funded, nonetheless MCAs faced stiff competition for posts (e.g. the NIHR funds around 250 ACFs and 100 clinical lecturer posts each year).[24] The following responses were typical:

> The biggest challenge is getting funding/grants/fellowships that are limited (MSR62ACF)

> There's no shortage of applicants; the problem is not having enough funding to go around. It's very difficult to get one of these posts. (MCA11ACF)

For peer review only - http://bmjopen.bmj.com/site/about/guidelines.xhtml

A particular concern of MCAs was how long it took them to qualify compared with colleagues who had chosen a purely clinical route:

> All my peers who started with me are looking at Consultant jobs now and I've still got almost three years left (MCA14CL).

Nevertheless, participants also valued the length of training, seeing it as an opportunity to accumulate skills and knowledge:

> The breadth of my clinical experience is a lot more than my clinical colleagues...I've seen more complications, more rare stuff, just because I've been around longer (MCA10CL).

Financial considerations:

The delays in clinical qualification challenged some NMAHPs' perception that pursuing a clinical academic career had 'no impact on medics' money':

> It has delayed my CCT [Certificate of Completion of Training] and substantially reduced my life-time earnings (MSR5CL).

This was exacerbated for MCAs who took parental leave and/or worked part-time:

> It really extends my training...that's five years of earning a Registrar level salary, rather than a Consultant one, and that's a big difference (MCA15CL).

For peer review only - http://bmjopen.bmj.com/site/about/guidelines.xhtml

In contrast, some NMAHPs who had progressed to high levels professionally before embarking on the clinical academic pathway, reported a drop in their earnings:

> When the medics do their on-call shifts they're not asked to downgrade in any way but…I was expected to work as a Band 5, and I've been Band 7 for twelve years. You're trying to push yourself forward academically but clinically it's not respected (CS2Nurse).

Again, contrasts were drawn with MCAs:

> You get the impression that it was much more set up for [MCAs] to be able to continue in research...they had an established career structure. Whereas in physiotherapy it's not the norm, it's massively the exception that involves you seeking a way of doing it. There isn't that pathway as part of the profession (CS14AHP).

Combining clinical practice and academic training

Both sets of participants expressed difficulties in convincing managers of the value of academic training. For example:

> The idea that you want to contribute in a different way is a bit like, 'Why? We need midwives at the frontline, not doing PhDs.' And that's such a short-sighted approach to the workforce. Because it's the opposite you see in the medics (CS8Midwife).

For peer review only

For peer review only - http://bmjopen.bmj.com/site/about/guidelines.xhtml

Despite this perception, MCAs also reported challenges through doing academic training in parallel with clinical training:

> We had to have around 25 hours [for] all the different study days. I really had to fight for them, I had to take annual leave, I had to reiterate again and again…Although there was lots of support from the academic side, there didn't really seem to be an understanding of what an academic trainee was (MCA13CL).

This suggests a lack of awareness around clinical academic careers within clinical settings, although according to the interviewees this varied between specialties. Nevertheless, it highlighted the need for improved communication between HEIs and NHS partners:

> You're working with [both] the hospital and the university so [it would be good for] the rota coordinators to be aware that you have academic commitments (MCA4ACF).

Despite the recommendation that 'both clinical and research training are properly ring-fenced',[3,p21] participants from both studies reported difficulties negotiating time for academic work. However, experiences varied depending on research funders. Participants funded by the NHS reported feeling reluctant to assert their right to time off for academic training, whereas those funded by NIHR seemed better able to assert themselves:

For peer review only - http://bmjopen.bmj.com/site/about/guidelines.xhtml

> That's the nature of the NIHR fellowship, it's 100% [whereas] when the department are paying some of the salary, they do expect some work for that (CS13 AHP).

Career pathway

While MCAs are either employed by a hospital trust with an honorary university contract or vice-versa,[1] there is currently no nationally agreed approach to contracts for NMAHPs. Consequently, some NMAHPs are employed by both organisations on separate contracts which often adversely affects their terms and conditions:

> Because it's not ingrained within our career structures, you're sort of chopped up into oh, well half of you will work in clinical practice and half will work in academia (CS9Nurse).

In addition, because PhDs are not part of the NMAHPs' career pathway, many were unable to retain their clinical post:

> I've had to step out of my area of expertise...I had to resign from my job that I'd been doing eighteen years to take on the PhD, which I wasn't impressed by...I asked to have a secondment, but they [NHS] won't let you (CS10AHP).

Some NMAHPs were anticipating problems in pursuing a clinical academic career post-PhD.

For peer review only - http://bmjopen.bmj.com/site/about/guidelines.xhtml

I can't see [employer] saying we value your research enough to change your job description…to a clinical academic. A lot of people in my position just go back to their clinical role (CS14AHP).

Despite CS14's research having had a major impact with changes implemented nationally, he still faced an uncertain future in relation to his specialist job role. Participants described issues at a structural level:

It's as if the higher-level managers are saying "yeah we need this", but they're not facilitating the managers to facilitate the clinicians. If we look at the medics, they've got these clinical academic posts firmly in place. And we need to have a firmly established pathway (CS15AHP).

However, both sets of participants expressed uncertainty about their post-doctoral prospects. Concerns around competition for limited funding and jobs were cited multiple times in both surveys and interviews:

My frustration [is that] the pathway is a pyramid therefore some people will not progress up (CS12AHP).

This 'pyramid' analogy was considered particularly problematic for female clinical academics:

The jobs are fewer and fewer the higher you get. A lot of women start off [clinical] academic and then leave along the way because there aren't enough jobs. Whereas if you're single and you've got no responsibilities you can move all over the country (MCA11ACF)

For peer review only

DISCUSSION

This is the first study to combine experiences of MCAs with NMAHPs pursuing a clinical academic pathway. Data revealed differences in demographics between the research populations. Most MCAs were younger, highlighting that for medical trainees combining research with their clinical practice is a normalised part of their career pathway from the beginning of their training. In contrast, gaining a PhD is not a usual part of NMAHPs' career ladder and, unlike the IAT pathway, NMAHPs embark on PhDs only after completing clinical training. This suggests not only that their potential is stymied, with a resultant delay to benefits emanating from their research,[11] but with fewer years left before retirement, the mantra of 'investment in research leaders of the future'[6] is questionable.

Rather than just focussing on barriers to clinical academic careers, this study further noted the importance of motivation,[5,7] particularly in terms of improving patient care. Findings revealed that early experiences of research were also influential, confirming previous studies that medical trainees who enjoyed research were likely to continue to be motivated,[4,,6,8,10] whereas negative experiences could result in people abandoning the clinical academic pathway. However, differences in provision between the two research populations reinforced recommendations that training in research design and evidence evaluation be made available to all healthcare professionals, starting at

For peer review only - http://bmjopen.bmj.com/site/about/guidelines.xhtml

undergraduate level.[6,25] If implemented, research could become normalised as part of NMAHPs' career pathways, in line with that of MCAs.

MCAs benefitted from dedicated academic time and financial support for career development through bursaries and fees. In contrast, NMAHPs often had to leave their post or reduce their hours to pursue clinical academic training. This confirms the findings of the Academy of Medical Sciences who highlight a 'critical need' to develop 'a sustainable infrastructure for research' as well as clearer academic career pathways for NMAHPs, 'supported by increased funding at both pre-and post-doctoral levels'.[6,P19]

Whilst MCAs' early experiences of disseminating research and grant applications potentially improved their career prospects, it resulted in delays to their clinical training with subsequent financial implications as well as presenting challenges in managing two sets of training simultaneously. This was a common theme across both data sets, with participants experiencing difficulties negotiating time for mandatory academic work with clinical managers. This echoes previous research suggesting a lack of understanding regarding the purpose and value of clinical academic careers and indicating differences in clinical and academic priorities of HEIs and NHS.[10] This study supports Westwood and colleagues' call for better communication to enable HEIs and NHS partners to work together to smooth the clinical academic pathway.[26]

For peer review only - http://bmjopen.bmj.com/site/about/guidelines.xhtml

Moreover, whilst MCAs had more job security and seemingly better career prospects, their progress, like NMAHPs', was dependent on securing grants and the availability of suitable positions. Participants from both groups likened the clinical academic pathway to a 'pyramid', with positions becoming scarcer and more competitive as they progressed which was considered particularly problematic for people who were limited geographically such as women with caring responsibilities. This study's findings therefore support recent recommendations to create more clinical academic posts, particularly at senior levels[6] in order to promote equality and inclusivity and build capacity in clinical research leadership. Furthermore, incorporating research time into clinical academic job descriptions would attract staff, increase job satisfaction and contribute to improved patient outcomes.[6]

Strengths and Limitations.

Adopting a mixed methods approach enhanced the research by using strengths of different approaches,[27] with similar themes identified across both data sets. Furthermore, individual interviews enabled a deeper understanding of clinical academic trainees' experiences, allowing exploration of nuanced meanings and apparent inconsistencies. These data can be difficult to capture in surveys or focus groups which are the usual methods for researching clinical academic careers.[4,5,8,10]

For peer review only - http://bmjopen.bmj.com/site/about/guidelines.xhtml

Although limited geographically, the study nevertheless encompassed two different HEIs and four large Trusts, revealing important differences and similarities between the clinical academic experiences of MCAs and NMAHPs as well as between two training sites. Future research should compare experiences of clinical academics from different geographical areas and also consider the perspectives of clinical managers.

CONCLUSION

Although both MCAs and NMAHPs are encouraged to pursue a clinical academic career, this research reveals inconsistencies in their respective career pathways. However, despite perceptions that MCAs have an easier route, this research has revealed obstacles in terms of financial penalisation and difficulties in climbing the clinical academic career ladder for both sets of participants in this study. These need to be addressed at structural level to enable clinical academics from all backgrounds to fully utilise their dual skills and talents and fulfil the NIHR aim to 'attract, develop and retain the best research professionals.'[1]

Author Contributions: All authors were involved in designing the study, data analysis and preparing this paper. The first author conducted all interviews.

Competing interests: All authors have completed the ICMJE uniform disclosure form at www.icmje.org/coi_disclosure.pdf and declare: all authors had financial support from the National Institute for Health Research (NIHR) Applied Research Collaboration for East Midlands (ARC

For peer review only - http://bmjopen.bmj.com/site/about/guidelines.xhtml

EM) for the submitted work; no financial relationships with any organisations that might have an interest in the submitted work in the previous three years; no other relationships or activities that could appear to have influenced the submitted work.

Funding: This work was supported by the National Institute for Health Research (NIHR) Applied Research Collaboration for East Midlands (ARC EM) grant number NIHR200171. The views expressed are those of the authors and not necessarily those of the NIHR or the Department of Health and Social Care.

Data sharing statement: Raw transcripts are held by the authors who will consider requests for further information.

REFERENCES

1 NIHR Trainees Coordinating Centre https://www.nihr.ac.uk/documents/explore-nihr/academy-programmes/NIHR%20Strategic%20Review%20of%20Training%202017.pdf (Accessed March 2020)

2 NIHR Integrated Academic Training Guide https://www.nihr.ac.uk/documents/iat-guide/22494 (Accessed April 2020)

3 NIHR https://www.nihr.ac.uk/explore-nihr/academy-programmes/hee-nihr-integrated-clinical-academic-programme.htm Accessed March 2020

For peer review only

4 Clough S, Fenton J, Harris-Joseph H, et al. What impact has the NIHR Academic Clinical Fellowship (ACF) scheme had on clinical academic careers in England over the last 10 years? A retrospective study. *BMJ Open*, 2017;*7*(6):e015722.

5 Goldacre MJ, Lambert TW, Goldacre R, et al. Career plans and views of trainees in the Academic Clinical Fellowship Programme in England. *Medical teacher* 2011;*33*(11):e637-e643.

6 The Academy of Medical Sciences, Transforming Health through innovation: Integrating the NHS and Academia 2020 https://acmedsci.ac.uk/file-download/23932583 (Accessed February 2020)

7 Ranieri V, Barratt H, Fulop N, Rees G. Factors that influence career progression among postdoctoral clinical academics: a scoping review of the literature. *BMJ Open*. 2016 Oct 1;6(10):e013523.

8 Lopes J, Ranieri V, Lambert T, et al. The clinical academic workforce of the future: a cross-sectional study of factors influencing career decision-making among clinical PhD students at two research-intensive UK universities. *BMJ Open* 2017;7.8:e016823

9 Stanley AG, Williams B, Gallen D. A novel foundation-year-two post in academic medicine. *JRSM*. 2005 Jan;98(1):10-3.

10 Green RH, Evans V, MacLeod S, et al. A qualitative study of the perspectives of key stakeholders on the delivery of clinical academic

For peer review only - http://bmjopen.bmj.com/site/about/guidelines.xhtml

training in the East Midlands. *JRSM open*. 2018 Jan;9(2):2054270417741843.

11 Author a

12 O'Brien BC, Harris IB, Beckman TJ, et al. Standards for reporting qualitative research: a synthesis of recommendations. *Academic Medicine*. 2014Sep1;89(9):1245-51.

13 Bristol Online Survey https://www.onlinesurveys.ac.uk

14 East Midlands Clinical Academic Practitioner Network http://www.clahrc-em.nihr.ac.uk/about/east-midlands-clinical-academic-practitioner-network

15 Ashworth K. Sampling. In Becker S, Bryman A, Ferguson H, eds. *Understanding Research for Social Policy and Social Work: Themes, methods and approaches, 2nd Edition*. Bristol:The Policy Press 2012:226-233

16 O'Connell Davidson J, Layder D, *Methods, Sex and Madness*, London, Routledge 1994

17 Health Research Authority https://www.hra.nhs.uk/ (Accessed February 2020)

18 Zhang Y, Wildemuth BM. Qualitative analysis of content. *Applications of social research methods to questions in information and library science*. 2009;308:319.

For peer review only - http://bmjopen.bmj.com/site/about/guidelines.xhtml

19 Srivastava P, Hopwood N. A practical iterative framework for qualitative data analysis. *International journal of qualitative methods*. 2009 Mar;8(1):76-84.

20 Bryman A, Becker S, In Becker S, Bryman A, Ferguson H, eds. *Understanding Research for Social Policy and Social Work: Themes, methods and approaches, 2nd Edition*. Bristol. The Policy Press.2012:128-133

21 Statista https://www.statista.com/statistics/698260/registered-doctors-united-kingdom-uk-by-gender-and-specialty/ (Accessed March 2020)

22 Jones D, *Health Service Journal Supplement*, https://www.hsj.co.uk/Uploads/y/r/i/Research--Impact-supplement-18th-Nov-2015.pdf (Accessed March 2020)

23 Royal College of Nursing 'Nursing through time' https://www.rcn.org.uk/-/media/royal-college-of-nursing/documents/publications/2017/april/pub-005736.pdf (Accessed March 2020)

24 NIHR https://www.nihr.ac.uk/explore-nihr/academy-programmes/integrated-academic-training.htm (Accessed April 2020)

25 Baltruks D, Callaghan P. https://councilofdeans.org.uk/wp-content/uploads/2018/08/Nursing-midwifery-and-allied-health-clinical-academic-research-careers-in-the-UK.pdf Accessed February 2020

For peer review only - http://bmjopen.bmj.com/site/about/guidelines.xhtml

26 Westwood G, Richardson A, Latter S, et al. Building clinical academic leadership capacity: sustainability through partnership. *Journal of Research in Nursing*, 2018:*23*(4)346-357.

27 Hammersley M, Troubles with triangulation. In MM Bergman, ed. *Advances in Mixed Methods Research*. London, Sage 2008:22-36.

For peer review only

For peer review only - http://bmjopen.bmj.com/site/about/guidelines.xhtml

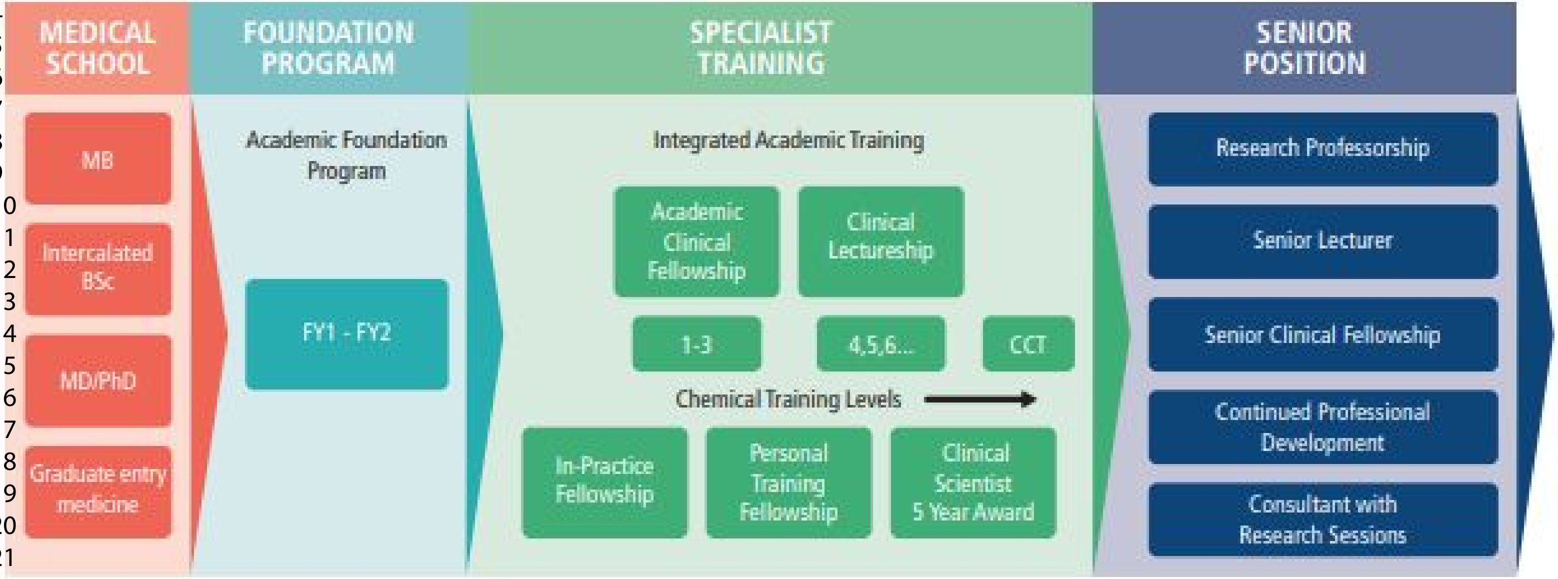

Figure 1. MCA progression chart (https://www.nihr.ac.uk/documents/iat-guide/22494)

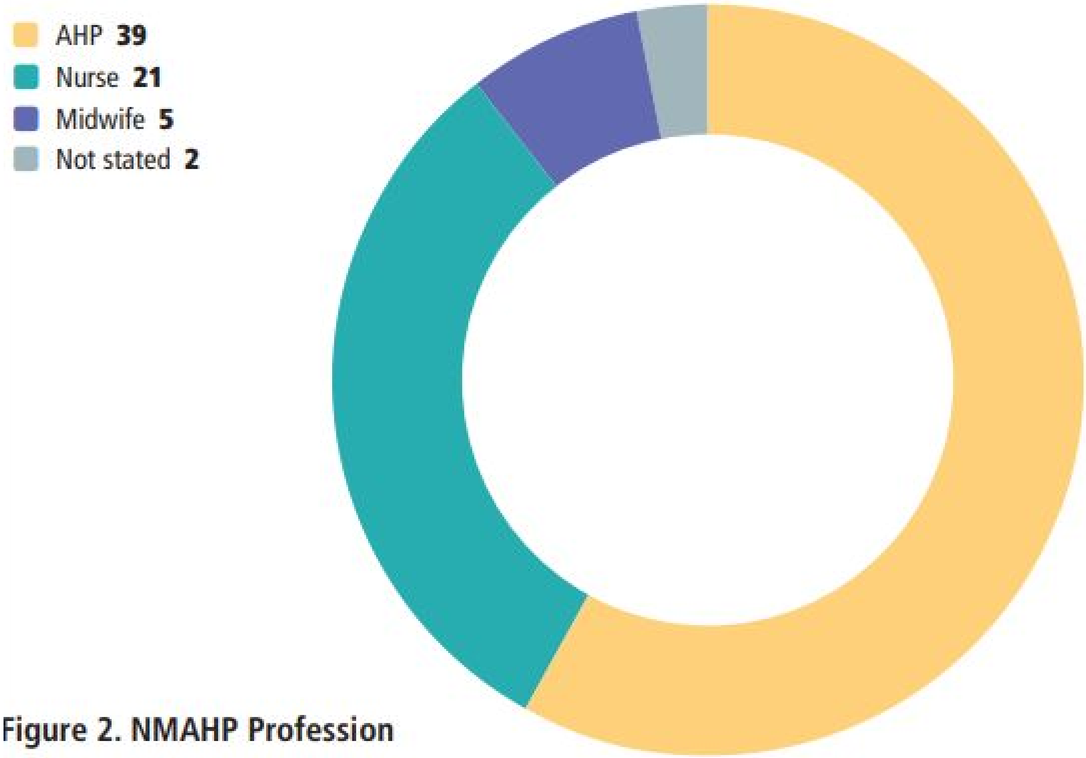

Figure 2. NMAHP Profession

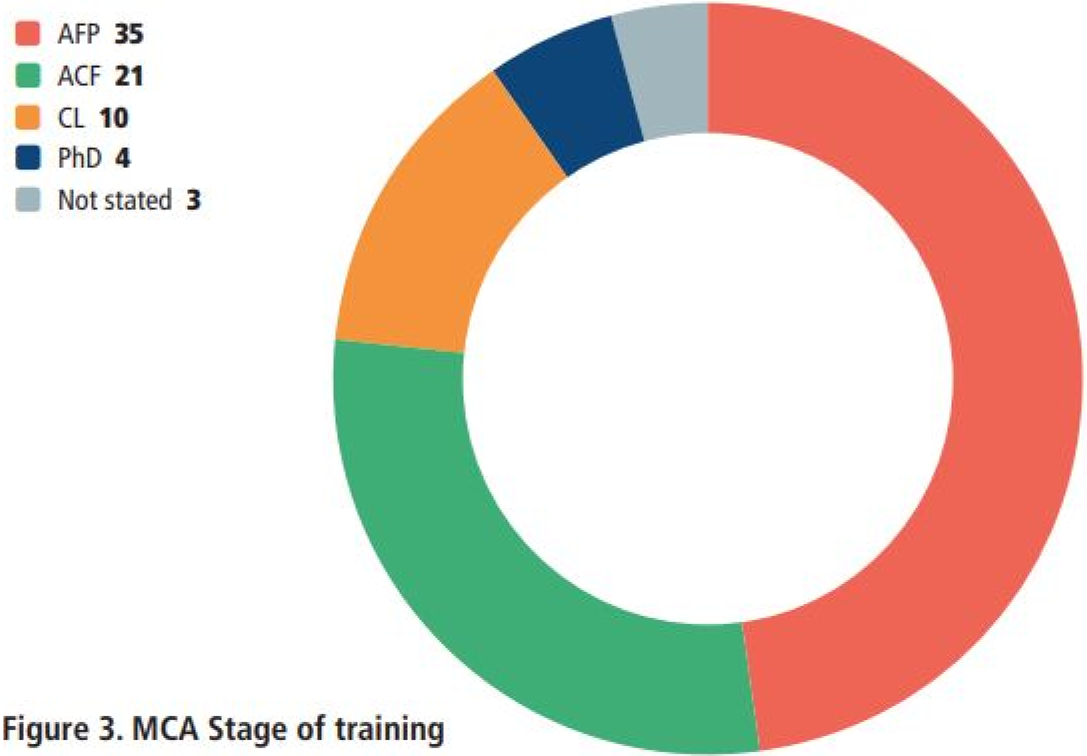

Figure 3. MCA Stage of training

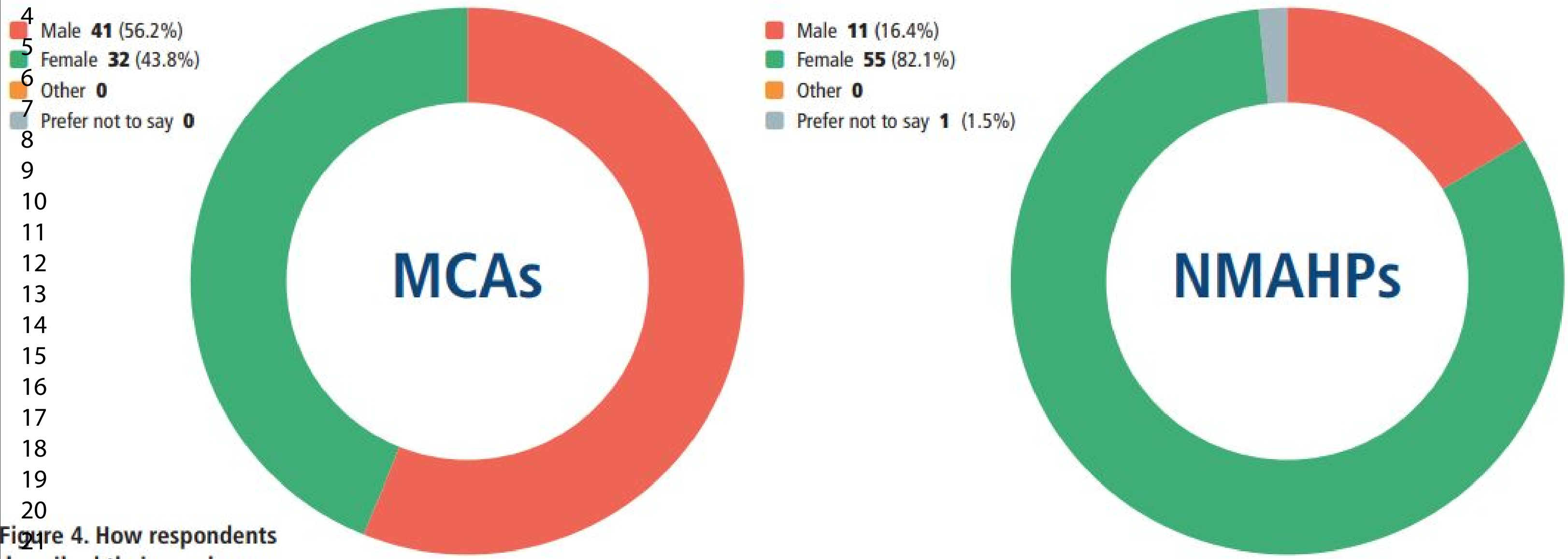

Figure 4. How respondents described their gender

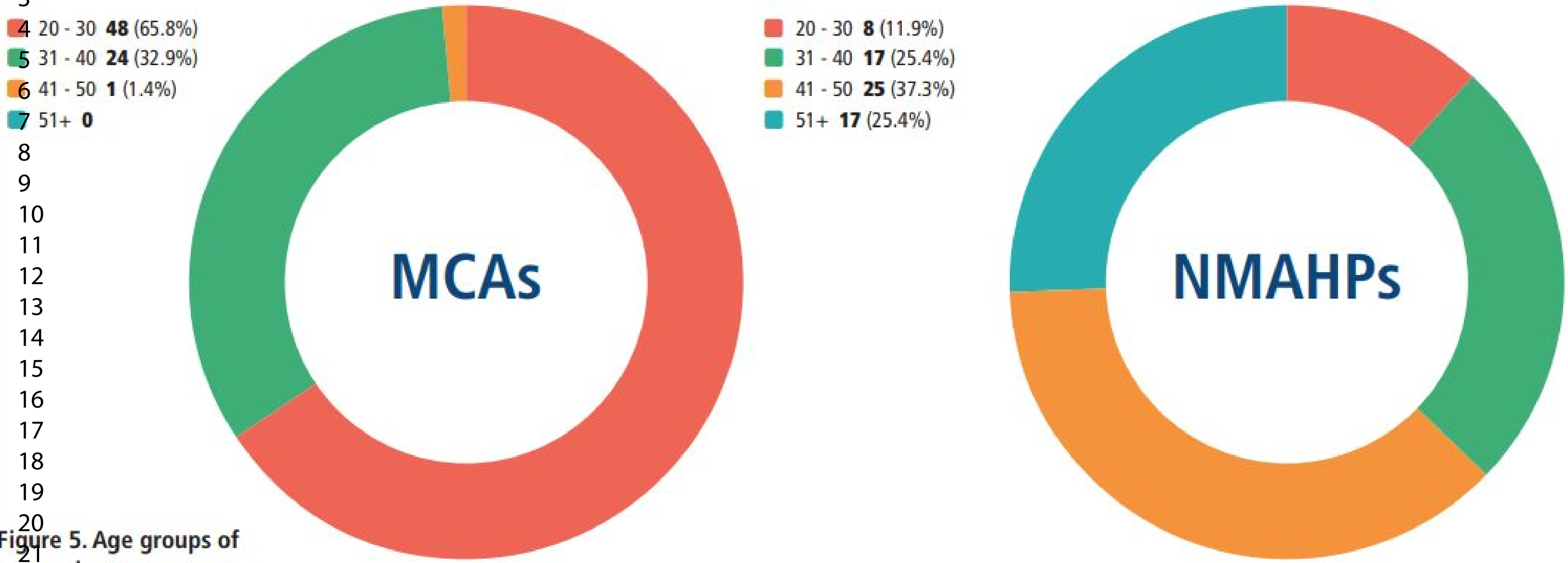

Figure 5. Age groups of respondents

Standards for Reporting Qualitative Research (SRQR)

O'Brien B.C., Harris, I.B., Beckman, T.J., Reed, D.A., & Cook, D.A. (2014). Standards for reporting qualitative research: a synthesis of recommendations. *Academic Medicine, 89(9)*, 1245-1251.

No. Topic	Item	Page
**Title and abstract**		
S1 Title	Concise description of the nature and topic of the study identifying the study as qualitative or indicating the approach (e.g., ethnography, grounded theory) or data collection methods (e.g., interview, focus group) is recommended	1
S2 Abstract	Summary of key elements of the study using the abstract format of the intended publication; typically includes objective, methods, results, and conclusions	2
**Introduction**		
S3 Problem formulation	Description and significance of the problem/phenomenon studied; review of relevant theory and empirical work; problem statement	3-5
S4 Purpose or research question	Purpose of the study and specific objectives or questions	4-5
**Methods**		
S5 Qualitative approach and research paradigm	Qualitative approach (e.g., ethnography, grounded theory, case study, phenomenology, narrative research) and guiding theory if appropriate; identifying the research paradigm (e.g., positivist, constructivist/interpretivist) is also recommended	5
S6 Researcher characteristics and reflexivity	Researchers' characteristics that may influence the research, including personal attributes, qualifications/experience, relationship with participants, assumptions, or presuppositions; potential or actual interaction between researchers' characteristics and the research questions, approach, methods, results, or transferability	8
S7 Context	Setting/site and salient contextual factors; rationale[a]	6
S8 Sampling strategy	How and why research participants, documents, or events were selected; criteria for deciding when no further sampling was necessary (e.g., sampling saturation); rationale[a]	7
S9 Ethical issues pertaining to human subjects	Documentation of approval by an appropriate ethics review board and participant consent, or explanation for lack thereof; other confidentiality and data security issues	8-9
S10 Data collection methods	Types of data collected; details of data collection procedures including (as appropriate) start and stop dates of data collection and analysis, iterative process, triangulation of sources/methods, and modification of procedures in response to evolving study findings; rationale[a]	6-7
S11 Data collection	Description of instruments (e.g., interview guides, questionnaires)	5-6,8

For peer review only

instruments and technologies	and devices (e.g., audio recorders) used for data collection; if/how the instrument(s) changed over the course of the study	
S12 Units of study	Number and relevant characteristics of participants, documents, or events included in the study; level of participation (could be reported in results)	10
S13 Data processing	Methods for processing data prior to and during analysis, including transcription, data entry, data management and security, verification of data integrity, data coding, and anonymization/deidentification of excerpts	9
S14 Data analysis	Process by which inferences, themes, etc., were identified and developed, including researchers involved in data analysis; usually references a specific paradigm or approach; rationale[a]	9
S15 Techniques to enhance trustworthiness	Techniques to enhance trustworthiness and credibility of data analysis (e.g., member checking, audit trail, triangulation); rationale[a]	9
**Results/Findings**		
S16 Synthesis and interpretation	Main findings (e.g., interpretations, inferences, and themes); might include development of a theory or model, or integration with prior research or theory	10-20
S17 Links to empirical data	Evidence (e.g., quotes, field notes, text excerpts, photographs) to substantiate analytic findings	10-20
**Discussion**		
S18 Integration with prior work, implications, transferability, and contribution(s) to the field	Short summary of main findings; explanation of how findings and conclusions connect to, support, elaborate on, or challenge conclusions of earlier scholarship; discussion of scope of application/generalizability; identification of unique contribution(s) to scholarship in a discipline or field	21-23
S19 Limitations	Trustworthiness and limitations of findings	23-24
**Other**		
S20 Conflicts of interest	Potential sources of influence or perceived influence on study conduct and conclusions; how these were managed	24
S21 Funding	Sources of funding and other support; role of funders in data collection, interpretation, and reporting	25

[a]The rationale should briefly discuss the justification for choosing that theory, approach, method, or technique rather than other options available, the assumptions and limitations implicit in those choices, and how those choices influence study conclusions and transferability. As appropriate, the rationale for several items might be discussed together.

For peer review only

East Midlands Clinical-Academic Practitioner Network

Clinical Academic Survey

Clinical academic survey

The East Midlands Clinical Academic Steering Group are hoping to demonstrate the impact of clinical academic training programmes so that future cohorts are able to benefit from the same opportunities that you have had.

With this in mind, we would be extremely grateful if you could take a few minutes to complete this questionnaire in Part 1 and also, if possible, to answer the additional questions in Part 2 (approx. 10 minutes). Part 3 is for people who are currently on a clinical academic training programme.

We appreciate that some work on this has already been undertaken by others, so if you have already taken part in a similar survey, we do not expect you to repeat the information.

Please be assured that all of your responses will be treated confidentially. Also, any quotes from the data that we may use when disseminating the results, will be anonymised.

All returned responses will be entered into a **prize draw** for a chance to **win a book of your choice up to a value of £50**. Please return the form by **20th April, 2018** and provide your name and email address here so that we can contact the prize winner.

For peer review only - http://bmjopen.bmj.com/site/about/guidelines.xhtml

Thank you very much in advance for your help. Good luck in the prize draw!

For peer review only

Part 1

Age group *Optional*

- 20-30
- 31-40
- 41-50
- 51+

How would you describe your gender?

- Male
- Female
- Other
- Prefer not to say

Current employment

- [ ] NHS
- [ ] University
- [ ] Both NHS and University
- [ ] Other employer

Please give the name(s) of your employer(s)

For peer review only - http://bmjopen.bmj.com/site/about/guidelines.xhtml

Please give your job title(s) and professional role (e.g assistant professor and nurse)

Are you employed:

- ◯ Full-time
- ◯ Part-time

At what level? (e.g. AFC Band or HEI scale)

For peer review only - http://bmjopen.bmj.com/site/about/guidelines.xhtml

Training programmes

Which of these clinical academic training programmes have you undertaken/are you undertaking? Please tick all that apply:

- ☐ Bronze fellowship
- ☐ Silver fellowship
- ☐ MARM
- ☐ NIHR funded MARM
- ☐ Masters in Clinical Research Studentship
- ☐ Other Masters programme
- ☐ Professional Doctorate
- ☐ NIHR funded PhD
- ☐ PhD not funded by NIHR
- ☐ Gold fellowship
- ☐ Other

Where did you undertake each training? (please list details for all courses that you have done) ✱ *Required*

How was your PhD funded?

If you selected Other, please specify: **Was it clinical academic training or another type of training?** Please provide details such as training provider; course completed/predicted completion date; qualification:

Which is the last training that you have done and when did it/will it finish? (month/year)

What were your reasons for stopping training at that point?

For current PhD students: When did you start your PhD? (month/year)

Is it NIHR funded?

- Yes
- No

If not, how is your PhD funded?

Where are you on the PhD journey?

For peer review only - http://bmjopen.bmj.com/site/about/guidelines.xhtml

- ☐ Year 1
- ☐ Year 2
- ☐ Year 3
- ☐ Year 4
- ☐ Year 5
- ☐ Year 6
- ☐ Thesis pending

What is your anticipated end date? (month/year)

For peer review only - http://bmjopen.bmj.com/site/about/guidelines.xhtml

Please give brief details below of what you have done as a consequence of your funding/training:

Have you changed your employment in any way? For example, new roles; grades etc?

Please describe ways in which your training has had an impact or changed your clinical practice:

Have you disseminated your research:

- ☐ Locally
- ☐ Nationally
- ☐ Both

Please provide details of how you have disseminated your research (e.g. publications, conference presentations/posters). If possible, please provide links or cut and paste details.

For peer review only - http://bmjopen.bmj.com/site/about/guidelines.xhtml

Have you had any successful grant applications or other collaborations?

- Yes
- No

If so, please provide details (e.g. the funding body, research project, grant reference (if any), and whether you were the lead or co-applicant)

Thank you for the answers you have provided so far. We would be grateful if you could also take a few minutes to complete some or all of the questions in part 2. Current clinical academics are also invited to complete part 3. Otherwise, please skip to the finish at the end of part 3 to submit your survey.

- Proceed to part 2
- Skip to part 3

For peer review only - http://bmjopen.bmj.com/site/about/guidelines.xhtml

Part 2

In this part of the questionnaire, we want to find out what the term 'clinical academic' means to you, and how the clinical academic training you have received has influenced your career.

The description 'clinical academic' is varied. We want to try to find a way to best explain what the role is. Please explain what you understand by the title 'clinical academic':

Do you consider yourself to be a clinical academic?

- Yes
- No

If so why? (for example, do you have funded research and clinical sessions?)

How has the clinical academic training you have received helped you in your career and/or clinical practice?

For peer review only - http://bmjopen.bmj.com/site/about/guidelines.xhtml

How did you overcome any challenges in achieving success and/or progressing to the next level in your clinical academic career?

What types of support did you find most helpful during your clinical academic training?

Have your goals changed during your training?

- Yes
- No

If so, please explain how and why your goals have changed:

For peer review only - http://bmjopen.bmj.com/site/about/guidelines.xhtml

Are there any resources that you found beneficial in developing your clinical academic role which you would be happy to share for the benefit of others?

These might include, for example, a job description or a report which was helpful in gaining the support of your managers for your training.

If you have any resources that you think might be useful, please send them as an attachment to the following email: diane.trusson@nottingham.ac.uk . Alternatively, please give link(s) to electronic resources here:

What advice would you give to people who are considering undertaking training and embarking on a clinical academic career?

Is there anything else that you want to add?

If you are currently on a clinical academic training programme (e.g. MRes/MARM/Bronze or Silver scholar/PhD/Post-doc), please complete the questions in Part 3. Otherwise, please press 'next' to finish on the final page.

For peer review only - http://bmjopen.bmj.com/site/about/guidelines.xhtml

Part 3. If you are currently on a clinical academic training programme e.g. MRes/MARM/Bronze or Silver scholar/PhD/Post-doc

Would you be interested in taking part in a focus group to discuss your experiences?

- Yes
- No

If so, please provide your contact details here:

We would also like to develop some case studies to demonstrate the impact that clinical academic training can have both in relation to your career and your clinical practice. Would you be willing to be interviewed about your experiences?

- Yes
- No

If so, please provide your contact details here:

For peer review only - http://bmjopen.bmj.com/site/about/guidelines.xhtml

Thank you for taking the time to complete this survey, your responses are very important to us.

For peer review only

For peer review only - http://bmjopen.bmj.com/site/about/guidelines.xhtml

Clinical Academic Survey for medics

Page 1: Clinical academic survey

Health Education England (HEE) PG Specialty School of Clinical Academic Training in the East Midlands

We are sending you this survey because we are interested in finding out more about your experiences of pursuing a clinical academic pathway. A similar survey has already been completed by nurses, midwives and allied health professionals in the East Midlands. The aim of this further study is to compare clinical academic experiences of different professions.

Completing the survey will take approximately 10 minutes and as a thank you for your time there is a chance to **win a £50 Amazon voucher**.

Please be assured that all of your responses will be treated confidentially. Also, any quotes from the data that we may use when disseminating the results, will be anonymised.

All returned responses will be entered into a **prize draw** for a chance to **win a £50 Amazon voucher!** Please return the form by midnight on **15th July, 2019** and provide your name and email address here so that we can contact the prize winner.

Thank you very much in advance for your help. Good luck in the prize draw!

For peer review only - http://bmjopen.bmj.com/site/about/guidelines.xhtml

Page 2: Part 1 - About you

Which age group are you in? *Optional*

- 20-30
- 31-40
- 41-50
- 51+

How would you describe your gender?

- Male
- Female
- Other
- Prefer not to say

How would you describe your ethnicity? (optional)

Current employment

- NHS
- University

For peer review only - http://bmjopen.bmj.com/site/about/guidelines.xhtml

Who are you employed by?

What is the name of the clinical practice where you are employed?

Which postgraduate specialty school are you a member of?

- ☐ Foundation School
- ☐ School of Anaesthesia
- ☐ School of Emergency Medicine
- ☐ Primary Care
- ☐ School of Medicine
- ☐ Obstetrics and Gynaecology
- ☐ Paediatrics
- ☐ Pathology
- ☐ Psychiatry
- ☐ Public Health
- ☐ Radiology
- ☐ Surgery

Please tell us the name of your clinical training programme

For peer review only - http://bmjopen.bmj.com/site/about/guidelines.xhtml

Page 3: Do you have a PhD/MD or are you studying for one?

If you are studying for a PhD, which University is your PhD affiliated with?

- ☐ University of Leicester
- ☐ University of Nottingham
- ☐ Other

Please give the name of University that your PhD is affiliated with

For current PhD/MD students: When did you start your PhD/MD? (month/year)

Who is funding your PhD?

Please give details of your PhD funding

Where are you on the PhD/MD journey?

- ☐ Year 1

For peer review only - http://bmjopen.bmj.com/site/about/guidelines.xhtml

- ☐ Year 2
- ☐ Year 3
- ☐ Year 4
- ☐ Year 5
- ☐ Year 6
- ☐ Submitted but not awarded

What is your anticipated submission date? (month/year)

For peer review only - http://bmjopen.bmj.com/site/about/guidelines.xhtml

Page 4

Please give brief details below of what you have done as part of your academic training:

Please describe ways in which your academic training has improved your skills as a clinician

Have you presented your research:

- ☐ Locally
- ☐ Nationally
- ☐ Internationally
- ☐ All the above

Please provide details of how you have disseminated your research (e.g. publications, conference presentations/posters). If possible, please provide links or cut and paste details.

Have you had any successful grant applications?

For peer review only - http://bmjopen.bmj.com/site/about/guidelines.xhtml

- Yes
- No

If so, please provide details (e.g. the funding body, research project, grant reference (if any), and whether you were the lead or co-applicant)

For peer review only

For peer review only - http://bmjopen.bmj.com/site/about/guidelines.xhtml

Page 5: Part 2. Becoming a clinical academic

In this part of the questionnaire, we want to find out what the term ‘clinical academic’ means to you, and how the clinical academic training you have received has influenced your career.

The description ‘clinical academic’ is varied. We want to try to find a way to best explain what the role is. Please explain what you understand by the title ‘clinical academic’:

Do you consider yourself to be a clinical academic?

- Yes
- No

If so why? (for example, do you have funded research and clinical sessions?)

How has your academic training helped you in your career? (if applicable)

For peer review only - http://bmjopen.bmj.com/site/about/guidelines.xhtml

If applicable, how did you overcome any challenges in achieving success and/or progressing to the next level in your clinical academic career?

What types of support did you find most helpful during your clinical academic training?

Do you anticipate any challenges in pursuing a clinical academic career? For example if you want to take breaks for parental leave or work part time in the future?

Have your goals changed during your training?

- Yes
- No

If so, please explain how and why your goals have changed:

For peer review only - http://bmjopen.bmj.com/site/about/guidelines.xhtml

Page 6

Are there any resources that you found beneficial in developing your clinical academic role which you would be happy to share for the benefit of others?

These might include, for example, websites where you have found useful advice and information.

If you have any resources that you think might be useful, please send them as an attachment to the following email: diane.trusson@nottingham.ac.uk . Alternatively, please give link(s) to electronic resources here:

What advice would you give to people who are considering a clinical academic career?

Is there anything else that you want to add?

For peer review only - http://bmjopen.bmj.com/site/about/guidelines.xhtml

Page 7: Part 3. Would you be willing to talk about your experiences?

Would you be interested in taking part in a focus group to discuss your experiences?

- Yes
- No

If so, please provide your contact details here:

We would also like to develop some case studies to demonstrate the impact that undertaking a clinical academic pathway can have both in relation to your career and your clinical practice. Would you be willing to be interviewed about your experiences?

- Yes
- No

If so, please provide your contact details here:

For peer review only - http://bmjopen.bmj.com/site/about/guidelines.xhtml

Page 8: Thank you for taking the time to complete this survey, your responses are very important to us.

Key for selection options

6.a - Who are you employed by?

University of Leicester
University of Nottingham
University Hospitals of Leicester
Nottingham University Hospitals
Partnership Trust
Other

8.a - Who is funding your PhD?

NIHR
Wellcome Trust
Medical Research Council (MRC)
Other

For peer review only

BMJ Open

A multi-methods study comparing the experiences of medical clinical academics with nurses, midwives and allied health professionals pursuing a clinical academic career.

Journal:	*BMJ Open*
Manuscript ID	bmjopen-2020-043270.R1
Article Type:	Original research
Date Submitted by the Author:	21-Jan-2021
Complete List of Authors:	Trusson, Diane; University of Nottingham, School of Medicine. NIHR Applied Research Collaboration East Midlands (ARC EM)<br>Rowley, Emma; University of Nottingham, School of Medicine. National Institute for Health Research (NIHR) Applied Research Collaboration East Midlands (ARC EM)<br>Barratt, Jonathan; University of Leicester College of Medicine Biological Sciences and Psychology
**Primary Subject Heading**:	Medical education and training
Secondary Subject Heading:	Qualitative research
Keywords:	EDUCATION & TRAINING (see Medical Education & Training), MEDICAL EDUCATION & TRAINING, QUALITATIVE RESEARCH

SCHOLARONE™
Manuscripts

I, the Submitting Author has the right to grant and does grant on behalf of all authors of the Work (as defined in the below author licence), an exclusive licence and/or a non-exclusive licence for contributions from authors who are: i) UK Crown employees; ii) where BMJ has agreed a CC-BY licence shall apply, and/or iii) in accordance with the terms applicable for US Federal Government officers or employees acting as part of their official duties; on a worldwide, perpetual, irrevocable, royalty-free basis to BMJ Publishing Group Ltd ("BMJ") its licensees and where the relevant Journal is co-owned by BMJ to the co-owners of the Journal, to publish the Work in this journal and any other BMJ products and to exploit all rights, as set out in our licence.

The Submitting Author accepts and understands that any supply made under these terms is made by BMJ to the Submitting Author unless you are acting as an employee on behalf of your employer or a postgraduate student of an affiliated institution which is paying any applicable article publishing charge ("APC") for Open Access articles. Where the Submitting Author wishes to make the Work available on an Open Access basis (and intends to pay the relevant APC), the terms of reuse of such Open Access shall be governed by a Creative Commons licence – details of these licences and which Creative Commons licence will apply to this Work are set out in our licence referred to above.

Other than as permitted in any relevant BMJ Author's Self Archiving Policies, I confirm this Work has not been accepted for publication elsewhere, is not being considered for publication elsewhere and does not duplicate material already published. I confirm all authors consent to publication of this Work and authorise the granting of this licence.

For peer review only

A multi-methods study comparing the experiences of medical clinical academics with nurses, midwives and allied health professionals pursuing a clinical academic career.

Author names

Diane Trusson, Emma Rowley and Jonathan Barratt

Correspondence address: Dr Diane Trusson, NIHR Applied Research Collaboration East Midlands (ARC EM), Institute of Mental Health, University of Nottingham Innovation Park, Triumph Road, Nottingham, NG7 2TU. Email: Diane.Trusson@nottingham.ac.uk; Phone: 0115 7484249

Diane Trusson PhD: NIHR Applied Research Collaboration East Midlands (ARC EM); University of Nottingham School of Medicine, UK. Orcid ID: 0000-0002-6995-1192

Emma Rowley PhD: NIHR Applied Research Collaboration East Midlands (ARC EM); University of Nottingham School of Medicine, UK. Orcid: 0000-0002-6668-1350

Jonathan Barratt PhD FRCP: University of Leicester and Leicester General Hospital, Leicester, UK. Orcid ID: 0000-0002-9063-7229

Key words: Clinical Academic careers; Medical Clinical Academics; Nurses, midwives and AHPs; Multi-methods; Interviews

Word count: 4202

For peer review only

For peer review only - http://bmjopen.bmj.com/site/about/guidelines.xhtml

ABSTRACT:

Objectives: This study aimed to compare experiences of medical clinical academics (MCAs) with those of nurses, midwives, and allied health professionals (NMAHPs) pursuing a clinical academic career.

Design: A multi-methods approach was used to elicit qualitative data. Both sets of participants completed similar online surveys followed by in-depth interviews to explore emerging themes.

Setting: The research was conducted in the East Midlands of England, encompassing two HEIs and four NHS Trusts.

Participants: Surveys were completed by 67 NMAHPs and 73 MCA trainees. Sixteen participants from each group were interviewed following a similar interview schedule.

Results: The survey data revealed notable differences in demographics of the two study populations, reflecting their different career structures. MCAs were younger and they all combined clinical and academic training, lengthening the time before qualification. In contrast, most NMAHPs had been in their clinical post for some years before embarking on a clinical academic pathway. Both routes had financial and personal repercussions and participants faced similar obstacles. However, there was also evidence of wide-ranging benefits from combining clinical and academic roles.

Conclusions: Variations in experiences between the two study populations highlight a need for a clear academic pathway for all health professionals,

For peer review only

as well as sufficient opportunities post-PhD to enable clinical academics to fully utilise their dual skills.

STRENGTHS AND LIMITATIONS:

- This is the first study to compare experiences of medical clinical academics with those of nurses, midwives and AHPs pursuing a clinical academic career.
- A multi-methods approach enabled both breadth and depth of data to be gathered for comparison purposes.
- The study was limited to one geographical area.

INTRODUCTION

Recognising the value of supporting clinicians to do research in their area of expertise, the National Institute for Health Research (NIHR) provides a range of schemes to encourage aspiring clinical academics.[1] For medical clinical academics (MCAs), the academic career path can begin at medical school with an intercalated degree or Bachelor of Medicine PhD programme, alongside other opportunities to be involved in research. Academic foundation programme (AFP) posts funded by Health Education England (HEE), offer protected academic time during foundation year 2. The NIHR Integrated Academic Training Pathway supports academic clinical fellowships (ACFs) enabling academic training alongside specialty training with the aim of supporting entry onto a PhD programme, doctoral research fellowships and Clinical Lectureships (CLs) enabling postdoctoral clinicians to split their time equally between clinical and academic training.[2] (See figure 1).

For peer review only - http://bmjopen.bmj.com/site/about/guidelines.xhtml

[Insert figure 1 here]

In parallel, the HEE and NIHR Integrated Clinical Academic (ICA) Programme provides research training awards for healthcare professionals (excluding doctors and dentists) wanting to develop careers combining clinical research and research leadership with continued clinical practice and professional development (see table 1).[3] In contrast to MCAs, the NMAHP clinical academic pathway awards can only be pursued post-graduation and upon completion of clinical preceptorship.

Internships	Masters' in Clinical Research Studentships	Clinical Doctoral Research Fellowships	Clinical Lectureships	Senior Clinical Lectureships
'A pre-masters clinical research taster'	'An introduction to clinical research theory and practice'	'Obtain a PhD by research whilst still developing clinical skills'	'Combine post-doctoral research in an academic position with continued clinical practice'	Combine research and research leadership in a senior academic position with continued clinical practice'

Table 1: HEE/NIHR Integrated Clinical Academic Programme

For both professional groups, benefits of pursuing a clinical academic career include intellectual stimulation and improved career prospects[4,5] as well as wide-ranging benefits for patient care.[6] Studies also identify challenges, including unsupportive work environments, competing pressures, scarcity of funding and senior academic appointments and difficulty achieving a good work/life balance.[7,8] Previous studies have focussed either on MCAs,[9,10,11] or on NMAHPs.[12] However, it remains unclear how the benefits and challenges associated with the clinical academic pathway compare between MCAs and NMAHPs.

For peer review only - http://bmjopen.bmj.com/site/about/guidelines.xhtml

In addition, concerns about a ‘decline in the capacity of NHS staff to undertake, or even engage with research’[6,p6] as well as rates of attrition along the clinical academic career trajectory, particularly of women,[6,13] have made it increasingly important to identify factors that encourage and enable continuation to higher levels of clinical academia.[7]

This paper reports on data from two studies conducted in the East Midlands: one with NMAHPs, and one with MCAs. The aim is to compare experiences of pursuing a clinical academic career between the two study populations.

METHODS

This manuscript has been prepared according to the Standards for Reporting Qualitative Research[14](see online supplementary file 1).

Study design

A qualitative methodology was used for this study in order to prioritise individual perspectives and allow themes to arise from the data.[15] The study had two data collection components. Firstly, an online survey gathered descriptive data from a relatively large number of respondents at different stages along the clinical academic pathway. This was followed by semi-structured interviews to enable a deeper exploration of individual experiences which can be lost in focus group interviews for example.[10]

Recruitment

Participants were recruited from two main medical teaching HEIs and four large NHS Trusts in the East Midlands

For peer review only - http://bmjopen.bmj.com/site/about/guidelines.xhtml

Half of each survey's respondents indicated that their willingness to be interviewed. Quota sampling was used[15] to select 16 respondents from each study population, representing a range of demographics and stages of training. See tables 2 and 3 for interview sample characteristics:

NMAHP Case study number	Clinical role	Stage of study (at interview)	Age group	Gender
NMA1	Nurse	Thesis pending	20-30	Female
NMA2	Nurse	Year 2 PhD	41-50	Female
NMA3	Nurse	Year 4 PhD	41-50	Female
NMA4	Midwife	Post-doc	41-50	Female
NMA5	Nurse	Year 3 PhD	41-50	Female
NMA6	AHP	Year 4 PhD	41-50	Female
NMA7	AHP	Year 2 PhD	41-50	Female
NMA8	Midwife	Year 5 PhD	41-50	Female
NMA9	Nurse	Post-doc	31-40	Male
NMA10	AHP	Year 2 PhD	41-50	Female
NMA11	AHP	Year 1 PhD	41-50	Female
NMA12	AHP	Year 4 PhD	51+	Male
NMA13	AHP	Year 3 PhD	31-40	Male
NMA14	AHP	Year 3 PhD	41-50	Male
NMA15	AHP	Post-doc	51+	Male
NMA16	AHP	Year 1 PhD	31-40	Male

Table 2 NMAHP Interview sample characteristics

MCA Case study number	Clinical training programme (at interview)	Age group	Gender
Med1	ACF	31-40	Female
Med2	Clinical lecturer	31-40	Female
Med3	Year 3 PhD	20-30	Female
Med4	ACF	20-30	Male
Med5	AFP	20-30	Male
Med6	AFP	20-30	Male
Med7	Year 2 PhD	31-40	Male
Med8	ACF	31-40	Female
Med9	Year 4 PhD	31-40	Male
Med10	Clinical lecturer	31-40	Female
Med11	ACF	20-30	Male
Med12	Clinical lecturer	31-40	Female
Med13	Clinical lecturer	31-40	Female
Med14	Clinical lecturer	31-40	Male
Med15	Year 2 PhD	31-40	Female
Med16	ACF	31-40	Female

Table 3 MCA Interview sample characteristics

Data collection

For peer review only - http://bmjopen.bmj.com/site/about/guidelines.xhtml

An academic online survey platform (JISC Online Survey)[16] was used in each study (see online supplementary files 2-3).

The survey questions were developed with reference to previous literature[5,7,10] and aimed to build on existing knowledge regarding trainees' experiences of clinical academic training and their career aspirations. The interview guides were developed to allow deeper exploration of individual experiences, following themes which arose in the surveys.

Both sets of participants were given the same set of survey and interview questions with the exception of questions relating to their specific circumstances, such as asking for participants' medical specialty rather than job title.

Furthermore, the MCA survey, which was created after the NMAHPs' data had been analysed, contained an additional question about anticipating challenges, such as taking parental leave or working part-time in the future. In interviews, MCAs were asked for their perspectives on combining clinical academic careers with family life. These themes arose unsolicited in the NMAHPs' interviews and were considered important to pursue in a comparative study because of concerns around diversity, particularly at higher levels[13] and suggestions that difficulty in achieving a good work-life balance could be a barrier to clinical academic career progression.[7]

For peer review only

A link to the survey was distributed through a clinical academic network for NMAHPs[17]and a database of 263 MCA trainees. Both surveys were open for approximately 2 months. Potential participants had the opportunity to enter a prize draw as an incentive to participate.

Acknowledging clinical academics' limited availability, individual interviews were conducted at a time and place to suit the interviewee. This was usually their place of work or a public place (e.g. café). All interviews began by asking about their experiences of being a clinical academic, enabling the participant to prioritise what they considered to be the most salient aspects of their experiences. Although the interviews were flexible, a pre-prepared list of questions enabled responses to be compared within the sample and also between research populations.[18]

All interviews were digitally recorded with the participants' permission.

Research Team

The team consisted of a medical sociologist, a capacity development manager and a clinical academic from the respective study population (a nurse in the NMAHP study and a surgeon in the MCA study) to help tailor the survey and interview questions and facilitate participant recruitment. The first author who conducted all the interviews, was an experienced social researcher with no prior relationship with the participants.

Ethical considerations

Following Health Research Authority[19] guidance, this research was not submitted for Ethics Board approval because participants were recruited

For peer review only

For peer review only - http://bmjopen.bmj.com/site/about/guidelines.xhtml

by virtue of their participation in educational programmes, not their NHS status. Nevertheless, the research was conducted in accordance with the Declaration of Helsinki.[20] Any identifying information given in order to enter the prize draw and/or offer to be interviewed, was removed prior to analysis. Interviewees were informed about the purpose of the research, their right to withdraw from the study and how their (anonymised) data would be used. Recorded verbal consent was obtained prior to each interview. When interviewing in a public space, privacy was ensured to maintain confidentiality.

In addition, participants were assured that data would be stored confidentially on secure University systems.

Data processing

Demographic descriptive data from the surveys were summarized in graph form and free text responses were collated using JISC software.[16] Interviews were transcribed professionally with all identifying data removed prior to analysis. To ensure anonymity, NMAHP survey respondents are identified as NMASR1-SR67 or case study number (NMA1-16) plus their role. MCA survey respondents are identified by MedSR1-73, or case study number (Med1-16) with their stage of training. In addition, data extracts used in the discussion were sent to the relevant participants to confirm that they could not be identified by the contents.

For peer review only - http://bmjopen.bmj.com/site/about/guidelines.xhtml

Analysis:

A framework analysis was used as it is an effective way of reducing and summarizing large amounts of qualitative data. It is appropriate for this type of study where 'it is important to compare and contrast [textual] data by themes across many cases'.[21,p.6] The first author conducted the initial coding through an inductive approach involving multiple readings of the survey responses and interview transcripts. The resulting codes were compiled into a set of potential themes[22] which were discussed and agreed with the co-authors.

Rigour

Combining survey and interview data increased the credibility of the findings.[15] During the analysis process, all authors discussed and agreed the emerging themes to ensure reliability.[15]

Patient and public involvement

No traditional patient/public involvement was included in this study; however, the clinical members of the author team were able to act as sounding boards for their respective communities, to ensure data collection tools and plans were appropriate for the clinical communities being explored.

RESULTS

The surveys were completed by 67 NMAHPs and 73 MCAs who described their job role (figure 2) or stage of training (figure 3) as follows:

[Insert figures 2 and 3 here]

For peer review only - http://bmjopen.bmj.com/site/about/guidelines.xhtml

Gender:

[Insert figure 4 here]

Figure 4 reveals relatively equal numbers of male (41) and female (32) MCAs survey respondents, whereas female NMAHPs outnumbered their male counterparts by 5:1. These figures are representative of the gender divide for these occupations nationally.[23] However, it is worth noting that these data were gathered from MCAs at an early stage in their careers and 'although approximately 50% of pre-doctoral ACFs are female…the percentage of female fellows declines with increasing seniority of award'.[1.p1]

Age:

There were major differences between the two populations in terms of age of the survey respondents:

[Insert figure 5 here]

Figure 5 reveals substantially higher numbers of MCAs in the 20-30 age group compared with NMAHPs. This reflects the career structures of the two populations. With medical trainees being encouraged to undertake academic training in parallel with their clinical training, they would be more likely to fall into the 20-30 bracket.

Although the number of NMAHPs aged 51+ was not high, it contrasted with the lack of any MCAs in this age group. This is consistent with findings which revealed that NMAHPs tend to commence the clinical

For peer review only - http://bmjopen.bmj.com/site/about/guidelines.xhtml

academic pathway after establishing their careers whereas MCAs practising regionally who are 50+ are no longer on the training pathway; hence they are not captured in this dataset.

In the following section, the themes which arose in the data (see figure 6) will be discussed, with the exception of the theme 'combining clinical academia and family life' where the richness of the data was such that it will be reported in full separately.

[insert figure 6 here]

Embarking on a clinical academic career

In contrast to medical trainees who are encouraged to embark on the clinical academic pathway early in their career, academic training was a step-change in NMAHPs' career trajectory. This seemed to lead to a difference in attitudes towards participants in the two studies. For example, younger NMAHPs had to overcome certain prejudices:

> We've got this traditional sort of snobbery that you have to serve X amount of years before you are worthy to do academia (NMA8 Midwife, female).

This reveals how NMAHPs might be discouraged from pursuing a clinical academic career at an early stage, potentially holding talent back.[11]

Motivation

For peer review only - http://bmjopen.bmj.com/site/about/guidelines.xhtml

Both sets of participants described being motivated to do research by a drive to improve patient care:

> What got my brain ticking was why one patient presented a certain way, and not another patient with exactly the same disease. I always wanted to know the why (Med2 Clinical lecturer, female).

However, findings revealed the range of research made possible by approaching it from different perspectives. For example:

> We don't want to just hand [research] over to medics. Because they see the women who are ill or need help, but we also need to improve care for the women who go through a normal pathway (NMA4 Midwife, female).

This is supported by the Dean of the NIHR, Professor Dave Jones:

> We need to back away from the doctor-driven research narrative…[nurses, midwives and AHPs] are the experts in what they do – they must be supported to do research because they are the ones who are seeing the gaps, and who know the questions that must be answered.[24,p3]

Another motivating factor for many MCAs was their early experiences of research:

> When I was a medical student [I took] one year out to do full-time research and I loved it (Med9 PhD, male).

For peer review only - http://bmjopen.bmj.com/site/about/guidelines.xhtml

This indicates the benefit of encouraging research, starting at undergraduate level.[6] In contrast, NMAHPs' prior exposure to research training varied according to their profession. For example, research has been part of the curriculum for student physiotherapists for many years, whereas nurses have only comparatively recently been required to undertake degree-level training.[25] This may explain why AHPs outnumbered nurses in the NMAHP study although changes may occur as more nurses emerge from training having had research experience.

However, while NMAHPs described choosing their research topic, the data revealed inconsistencies in the opportunities for MCAs to pursue their own personal research interests. One participant described the process for assigning research topics:

> We all put our names in a hat...I ended up with my third or fourth choice (Med3 ACF, Female).

Although this practice was only reported by participants from one training provider, it had the potential to deter people from doing research. For example, one academic clinical fellow described being assigned a project which was not achievable in the 9 months available. Consequently, she had decided to focus solely on clinical training while her family were young.

> I haven't regretted doing it but…I haven't enjoyed it enough to keep going with it while I'm bringing up a family (Med1 ACF, female).

For peer review only - http://bmjopen.bmj.com/site/about/guidelines.xhtml

Similarly, several female NMAHPs described waiting until their children reached school age before embarking on a clinical academic pathway:

> My youngest daughter started school, so I had a bit of head space to think about where I wanted to take my career (NMA2 Nurse, female).

Comparisons with MCAs

NMAHPs expressed frustration at what they perceived to be an 'easier' route for MCAs:

> For them it's a perfectly normal part of your career to do a PhD...it's such a shame that the culture is slow to be absorbed into the nursing profession (NMA5 Nurse, female).

> A doctor I've been working with was doing an MD…He said it was more prestigious to do a PhD, so they've just swapped him. Do you know the pains that I went through to get PhD funding and you've just swapped? (NMA11 AHP, female).

This participant seemed particularly unhappy by what was perceived as unfair financial penalisation:

> [Doctors] are given 2-3 days a week to do research, plus travel and parking expenses. My department doesn't have a training budget anymore. Any extra training you get, you pay out of your

For peer review only - http://bmjopen.bmj.com/site/about/guidelines.xhtml

own pocket; you don’t get study leave, you have to take annual leave. It’s not equitable is it?’ (NMA11 AHP, female).

The benefits of this support were confirmed by MCAs:

I've been to lots of conferences…I've met a lot of influential people [its] really beneficial, for creating opportunities for my future (Med3 PhD, female)

Although the medical clinical academic route was better designed and funded, nonetheless MCAs faced stiff competition for posts (e.g. the NIHR funds around 250 ACFs and 100 clinical lecturer posts each year).[26] The following responses were typical:

The biggest challenge is getting funding/grants/fellowships that are limited (MedSR62 ACF, female)

There’s no shortage of applicants; the problem is not having enough funding to go around. It’s very difficult to get one of these posts. (Med11 ACF, male)

A particular concern of MCAs was how long it took them to qualify compared with colleagues who had chosen a purely clinical route:

All my peers who started with me are looking at Consultant jobs now and I’ve still got almost three years left (Med14 Clinical lecturer, male).

For peer review only

For peer review only - http://bmjopen.bmj.com/site/about/guidelines.xhtml

Nevertheless, participants also valued the length of training, seeing it as an opportunity to accumulate skills and knowledge:

> The breadth of my clinical experience is a lot more than my clinical colleagues...I've seen more complications, more rare stuff, just because I've been around longer (Med10 Clinical lecturer, female).

Financial considerations:

The delays in clinical qualification challenged some NMAHPs' perception that pursuing a clinical academic career had 'no impact on medics' money':

> It has delayed my CCT [Certificate of Completion of Training] and substantially reduced my life-time earnings (MedSR5 Clinical lecturer, female).

This was exacerbated for MCAs who took parental leave and/or worked part-time:

> It really extends my training...that's five years of earning a Registrar level salary, rather than a Consultant one, and that's a big difference (Med15 Clinical lecturer, female).

In contrast, some NMAHPs who had progressed to high levels professionally before embarking on the clinical academic pathway, reported a drop in their earnings:

> When the medics do their on-call shifts they're not asked to downgrade in any way but…I was expected to work as a Band 5,

For peer review only - http://bmjopen.bmj.com/site/about/guidelines.xhtml

> and I’ve been Band 7 for twelve years. You're trying to push yourself forward academically but clinically it's not respected (NMA2 Nurse, female).

Again, contrasts were drawn with MCAs:

> You get the impression that it was much more set up for [MCAs] to be able to continue in research...they had an established career structure. Whereas in physiotherapy it's not the norm, it's massively the exception that involves you seeking a way of doing it. There isn't that pathway as part of the profession (NMA14 AHP, male).

Combining clinical practice and academic training

Both sets of participants expressed difficulties in convincing managers of the value of academic training. For example:

> The idea that you want to contribute in a different way is a bit like, ‘Why? We need midwives at the frontline, not doing PhDs.’ And that’s such a short-sighted approach to the workforce. Because it’s the opposite you see in the medics (NMA8 Midwife, female).

Despite this perception, MCAs also reported challenges through doing academic training in parallel with clinical training:

> We had to have around 25 hours [for] all the different study days. I really had to fight for them, I had to take annual leave, I had to reiterate again and again…Although there was lots of support from

For peer review only - http://bmjopen.bmj.com/site/about/guidelines.xhtml

> the academic side, there didn't really seem to be an understanding of what an academic trainee was (Med13 Clinical lecturer, female).

This suggests a lack of awareness around clinical academic careers within clinical settings, although according to the interviewees this varied between specialties. Nevertheless, it highlighted the need for improved communication between HEIs and NHS partners:

> You're working with [both] the hospital and the university so [it would be good for] the rota coordinators to be aware that you have academic commitments (Med4 ACF male).

Despite the recommendation that 'both clinical and research training are properly ring-fenced',[3,p21] participants from both studies reported difficulties negotiating time for academic work. However, experiences varied depending on research funders. Participants funded by the NHS reported feeling reluctant to assert their right to time off for academic training, whereas those funded by NIHR seemed better able to assert themselves:

> That's the nature of the NIHR fellowship, it's 100% [whereas] when the department are paying some of the salary, they do expect some work for that (NMA13 AHP, male).

Career pathway

While MCAs are either employed by a hospital trust with an honorary university contract or vice-versa,[1] there is currently no nationally agreed

For peer review only - http://bmjopen.bmj.com/site/about/guidelines.xhtml

approach to contracts for NMAHPs. Consequently, some NMAHPs are employed by both organisations on separate contracts which often adversely affects their terms and conditions:

> Because it's not ingrained within our career structures, you're sort of chopped up into oh, well half of you will work in clinical practice and half will work in academia (NMA9 Nurse, male).

In addition, because PhDs are not part of the NMAHPs' career pathway, many were unable to retain their clinical post:

> I've had to step out of my area of expertise...I had to resign from my job that I'd been doing eighteen years to take on the PhD, which I wasn't impressed by...I asked to have a secondment, but they [NHS] won't let you (NMA10 AHP, female).

Some NMAHPs were anticipating problems in pursuing a clinical academic career post-PhD.

> I can't see [employer] saying we value your research enough to change your job description…to a clinical academic. A lot of people in my position just go back to their clinical role (NMA14 AHP, male).

Despite NMA14's research having had a major impact with changes implemented nationally, he still faced an uncertain future in relation to his specialist job role. Participants described issues at a structural level:

For peer review only - http://bmjopen.bmj.com/site/about/guidelines.xhtml

> It's as if the higher-level managers are saying "yeah we need this", but they're not facilitating the managers to facilitate the clinicians. If we look at the medics, they've got these clinical academic posts firmly in place. And we need to have a firmly established pathway (NMA15 AHP, male).

However, both sets of participants expressed uncertainty about their post-doctoral prospects. Concerns around competition for limited funding and jobs were cited multiple times in both surveys and interviews:

> My frustration [is that] the pathway is a pyramid therefore some people will not progress up (NMA12 AHP, male).

This 'pyramid' analogy was considered particularly problematic for female clinical academics:

> The jobs are fewer and fewer the higher you get. A lot of women start off [clinical] academic and then leave along the way because there aren't enough jobs. Whereas if you're single and you've got no responsibilities you can move all over the country (Med11 ACF, male).

DISCUSSION

This is the first study to combine experiences of MCAs with NMAHPs pursuing a clinical academic pathway. Data revealed differences in

For peer review only - http://bmjopen.bmj.com/site/about/guidelines.xhtml

demographics between the research populations. Most MCAs were younger, highlighting that for medical trainees combining research with their clinical practice is a normalised part of their career pathway from the beginning of their training. In contrast, gaining a PhD is not a usual part of NMAHPs' career ladder and, unlike the IAT pathway, NMAHPs embark on PhDs only after completing clinical training. This suggests not only that their potential is stymied, with a resultant delay to benefits emanating from their research,[12] but with fewer years left before retirement, the mantra of 'investment in research leaders of the future'[6] is questionable.

Rather than just focussing on barriers to clinical academic careers, this study further noted the importance of motivation,[5,7] particularly in terms of improving patient care. Findings revealed that early experiences of research were also influential, confirming previous studies that medical trainees who enjoyed research were likely to continue to be motivated,[4,6,8,10] whereas negative experiences could result in people abandoning the clinical academic pathway. However, differences in provision between the two research populations reinforced recommendations that training in research design and evidence evaluation be made available to all healthcare professionals, starting at undergraduate level.[6,27] If implemented, research could become normalised as part of NMAHPs' career pathways, in line with that of MCAs.

MCAs benefitted from dedicated academic time and financial support for career development through bursaries and fees. In contrast, NMAHPs

For peer review only - http://bmjopen.bmj.com/site/about/guidelines.xhtml

often had to leave their post or reduce their hours to pursue clinical academic training. This confirms the findings of the Academy of Medical Sciences who highlight a 'critical need' to develop 'a sustainable infrastructure for research' as well as clearer academic career pathways for NMAHPs, 'supported by increased funding at both pre-and post-doctoral levels'.[6,P19]

Whilst MCAs' early experiences of disseminating research and grant applications potentially improved their career prospects, it resulted in delays to their clinical training with subsequent financial implications as well as presenting challenges in managing two sets of training simultaneously. This was a common theme across both data sets, with participants experiencing difficulties negotiating time for mandatory academic work with clinical managers. This echoes previous research suggesting a lack of understanding regarding the purpose and value of clinical academic careers and indicating differences in clinical and academic priorities of HEIs and NHS.[10] This study supports Westwood and colleagues' call for better communication to enable HEIs and NHS partners to work together to smooth the clinical academic pathway.[28]

Moreover, whilst MCAs had more job security and seemingly better career prospects, their progress, like NMAHPs', was dependent on securing grants and the availability of suitable positions. Participants from both groups likened the clinical academic pathway to a 'pyramid', with positions becoming scarcer and more competitive as they progressed

For peer review only - http://bmjopen.bmj.com/site/about/guidelines.xhtml

which was considered particularly problematic for people who were limited geographically such as women with caring responsibilities. This study's findings therefore support recent recommendations to create more clinical academic posts, particularly at senior levels[6,13] in order to promote equality and inclusivity and build capacity in clinical research leadership. Furthermore, incorporating research time into clinical academic job descriptions would attract staff, increase job satisfaction and contribute to improved patient outcomes.[6]

Strengths and Limitations.

Combining survey and interview data enhanced the research, with similar themes identified across both data sets. Furthermore, individual interviews enabled a deeper understanding of clinical academic trainees' experiences, allowing exploration of nuanced meanings and apparent inconsistencies. These data can be difficult to capture in surveys or focus groups which are the usual methods for researching clinical academic careers.[4,5,8,10]

Although limited geographically, the study nevertheless encompassed two different HEIs and four large Trusts, revealing important differences and similarities between the clinical academic experiences of MCAs and NMAHPs as well as between two training sites. Suggestions for future research include comparing experiences of clinical academics from

For peer review only - http://bmjopen.bmj.com/site/about/guidelines.xhtml

different geographical areas and exploring the perspectives of clinical managers.

CONCLUSION

Although both MCAs and NMAHPs are encouraged to pursue a clinical academic career, this research reveals inconsistencies in their respective career pathways. However, despite perceptions that MCAs have an easier route, this research has revealed obstacles in terms of financial penalisation and difficulties in climbing the clinical academic career ladder for both sets of participants in this study.

Our research suggests that the NIHR could fulfil their aim to 'attract, develop and retain the best research professionals'[1] by providing more well-funded posts at senior level, research skills development in nursing and midwifery undergraduate courses, clearer run-through pathways in nursing/midwifery, and protected time for research within clinical contracts negotiated with clinical managers. This will enable clinical academics from all backgrounds to fully utilise their dual skills and talents.

Author Contributions: All authors (DT, ER and JB) contributed to the study design and data analysis. DT collected the data and drafted the manuscript as lead author. ER and JB contributed comments and edits to the manuscript. Final approval was given by all authors.

For peer review only - http://bmjopen.bmj.com/site/about/guidelines.xhtml

Competing interests: All authors have completed the ICMJE uniform disclosure form at www.icmje.org/coi_disclosure.pdf and declare: all authors had financial support from the National Institute for Health Research (NIHR) Applied Research Collaboration East Midlands (ARC EM) for the submitted work; no financial relationships with any organisations that might have an interest in the submitted work in the previous three years; no other relationships or activities that could appear to have influenced the submitted work.

Funding: This work was supported by the National Institute for Health Research (NIHR) Applied Research Collaboration East Midlands (ARC EM) grant number NIHR200171. The views expressed are those of the authors and not necessarily those of the NIHR or the Department of Health and Social Care.

Data sharing statement: Raw transcripts are held by the authors who will consider requests for further information.

Acknowledgments: The authors wish to thank all of the survey respondents and interview participants in this study. Also, thanks go to Dr Louise Bramley for her contributions, and to the reviewers' for their valuable feedback on a previous version of the manuscript.

For peer review only

REFERENCES

1 NIHR Trainees Coordinating Centre [Accessed Jan 2021] Available from: https://www.nihr.ac.uk/documents/explore-nihr/academy-programmes/NIHR%20Strategic%20Review%20of%20Training%202017.pdf

2 NIHR Integrated Academic Training Guide [Accessed Jan 2021] Available from: https://www.nihr.ac.uk/documents/iat-guide/22494

3 HEE-NIHR Integrated Clinical Academic Programme [Accessed Jan 2021] Available from: https://www.nihr.ac.uk/explore-nihr/academy-programmes/hee-nihr-integrated-clinical-academic-programme.htm

4 Clough S, Fenton J, Harris-Joseph H, et al. What impact has the NIHR Academic Clinical Fellowship (ACF) scheme had on clinical academic careers in England over the last 10 years? A retrospective study. *BMJ Open*, 2017;*7*(6):e015722.

5 Goldacre MJ, Lambert TW, Goldacre R, et al. Career plans and views of trainees in the Academic Clinical Fellowship Programme in England. *Medical teacher* 2011;*33*(11):e637-e643.

6 The Academy of Medical Sciences, Transforming Health through innovation: Integrating the NHS and Academia 2020. [Accessed January 2021] Available from: https://acmedsci.ac.uk/file-download/23932583

For peer review only - http://bmjopen.bmj.com/site/about/guidelines.xhtml

7 Ranieri V, Barratt H, Fulop N, Rees G. Factors that influence career progression among postdoctoral clinical academics: a scoping review of the literature. *BMJ Open*. 2016 Oct 1;6(10):e013523.

8 Lopes J, Ranieri V, Lambert T, et al. The clinical academic workforce of the future: a cross-sectional study of factors influencing career decision-making among clinical PhD students at two research-intensive UK universities. *BMJ Open* 2017;7.8:e016823

9 Stanley AG, Williams B, Gallen D. A novel foundation-year-two post in academic medicine. *JRSM*. 2005 Jan;98(1):10-3.

10 Green RH, Evans V, MacLeod S, et al. A qualitative study of the perspectives of key stakeholders on the delivery of clinical academic training in the East Midlands. *JRSM open*. 2018 Jan;9(2):2054270417741843.

11 Trusson D, Rowley E and Barratt J. Anticipating, experiencing, and overcoming challenges in clinical academic training. *British Journal of Healthcare Management* 2020 ISN 1358-0574 *(In press)*

12 Trusson D, Rowley E and Bramley L. A mixed-methods study of challenges and benefits of clinical academic careers for nurses, midwives, and allied health professionals *BMJ Open* 2019: http://dx.doi.org/10.1136/bmjopen-2019-030595

13 Vassie C, Smith S, Leedham-Green K. Factors impacting on retention, success and equitable participation in clinical academic careers: a scoping

For peer review only - http://bmjopen.bmj.com/site/about/guidelines.xhtml

review and meta-thematic synthesis. *BMJ Open* 2020 Mar1;10(3):e033480.

14 O'Brien BC, Harris IB, Beckman TJ, et al. Standards for reporting qualitative research: a synthesis of recommendations. *Academic Medicine*. 2014Sep1;89(9):1245-51.

15 Becker S, Bryman A, Ferguson H, eds. *Understanding Research for Social Policy and Social Work: Themes, methods and approaches, 2nd Edition*. Bristol:The Policy Press 2012

16 Jisc Online Surveys (formerly Bristol Online Surveys) [Accessed Jan 2021]. Available from: https://www.onlinesurveys.ac.uk/

17 East Midlands Clinical Academic Practitioner Network. NIHR Applied Research Collaboration East Midlands [Accessed Jan 2021] Available from: http://arc-em.nihr.ac.uk/about/east-midlands-clinical-academic-practitioner-network

18 O'Connell Davidson J, Layder D, *Methods, Sex and Madness*, London, Routledge 1994

19 Health Research Authority https://www.hra.nhs.uk/ (Accessed February 2020)

20 MedEdPublish – Policy on protection of research participants. Version 01: June 2019 [Accessed January 2021] Available from: https://www.mededpublish.org/Policies/Protection-of-Research-Participants

For peer review only - http://bmjopen.bmj.com/site/about/guidelines.xhtml

21 Gale NK, Heath G, Cameron E, Rashid S, Redwood S. Using the framework method for the analysis of qualitative data in multi-disciplinary health research. *BMC medical research methodology*. 2013 Dec;13(1):1-8

22 Braun V, Clarke V. Using thematic analysis in psychology. *Qualitative research in psychology*. 2006 Jan 1;3(2):77-101.

23 Statista 'Number of registered doctors in the UK in 2020 by gender and specialty' [Accessed March 2020] Available from:

https://www.statista.com/statistics/698260/registered-doctors-united-kingdom-uk-by-gender-and-specialty/

24 Trueland, J 'Birth of a new breed' *Health Service Journal 'Knowledge'*, [Cited December 2020] Available from https://www.hsj.co.uk/hsj-knowledge/birth-of-a-new-breed/7000387.article 18 Nov 2015

25 Thomas BG, Rosser E. Responsibility, research and reasoning: nursing through 70 years of the NHS. *British Journal of Nursing*. 2018 Jul 12;27(13):784-6.

26 NIHR Integrated Academic Programme [Accessed December 2020]

https://www.nihr.ac.uk/explore-nihr/academy-programmes/integrated-academic-training.htm

27 Baltruks D, Callaghan P. Nursing, midwifery and allied health clinical academic research careers in the UK. Councils of Deans of Health 2018 [Accessed January 2021] Available from:

https://councilofdeans.org.uk/wp-content/uploads/2018/08/Nursing-

For peer review only - http://bmjopen.bmj.com/site/about/guidelines.xhtml

midwifery-and-allied-health-clinical-academic-research-careers-in-the-UK.pdf

28 Westwood G, Richardson A, Latter S, et al. Building clinical academic leadership capacity: sustainability through partnership. *Journal of Research in Nursing*, 2018:*23*(4)346-357.

Figures:

- Figure 1. Medical Clinical Academic progression
- Figure 2. NMAHP's profession
- Figure 3. MCA stage of training
- Figure 4. How participants described their gender
- Figure 5. Age groups of survey respondents
- Figure 6. Coding tree

For peer review only

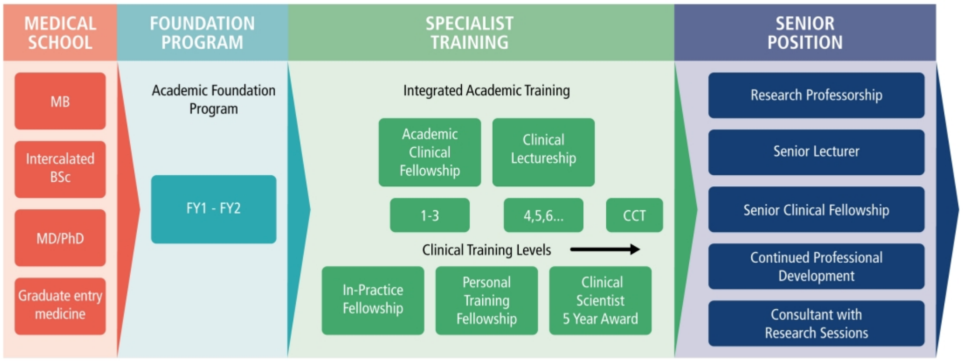

Figure 1. MCA progression chart (https://www.nihr.ac.uk/documents/iat-guide/22494)

Figure 1. MCA progression chart

762x355mm (100 x 100 DPI)

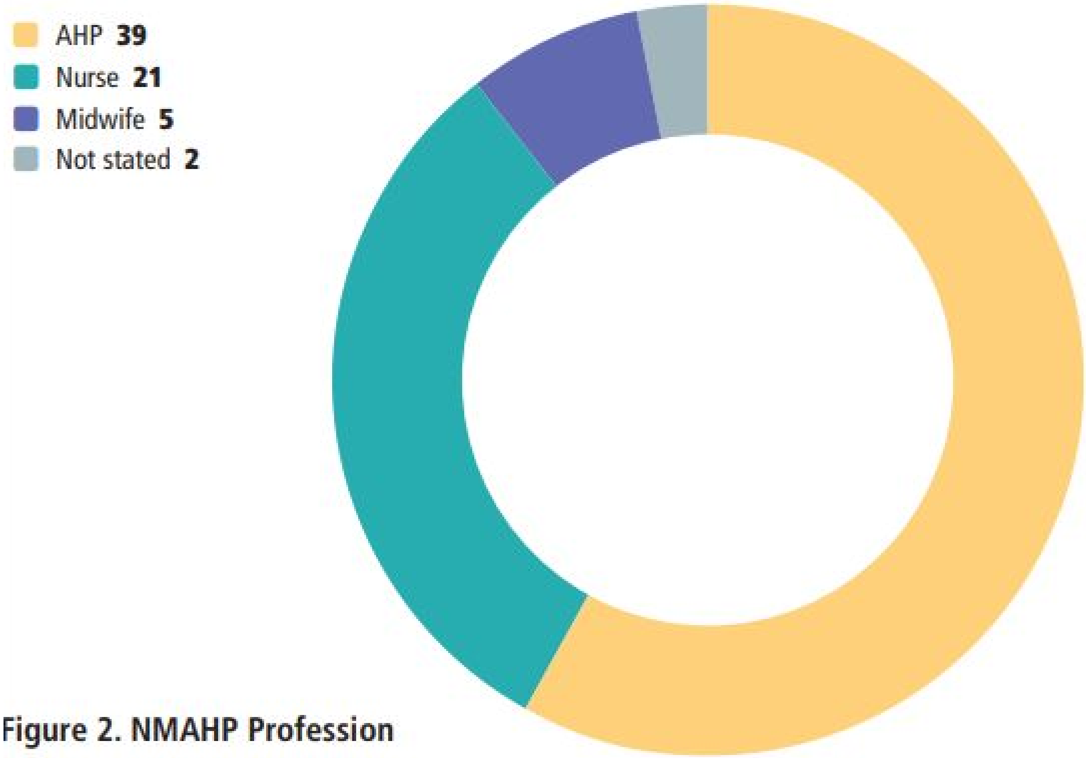

Figure 2. NMAHP Profession

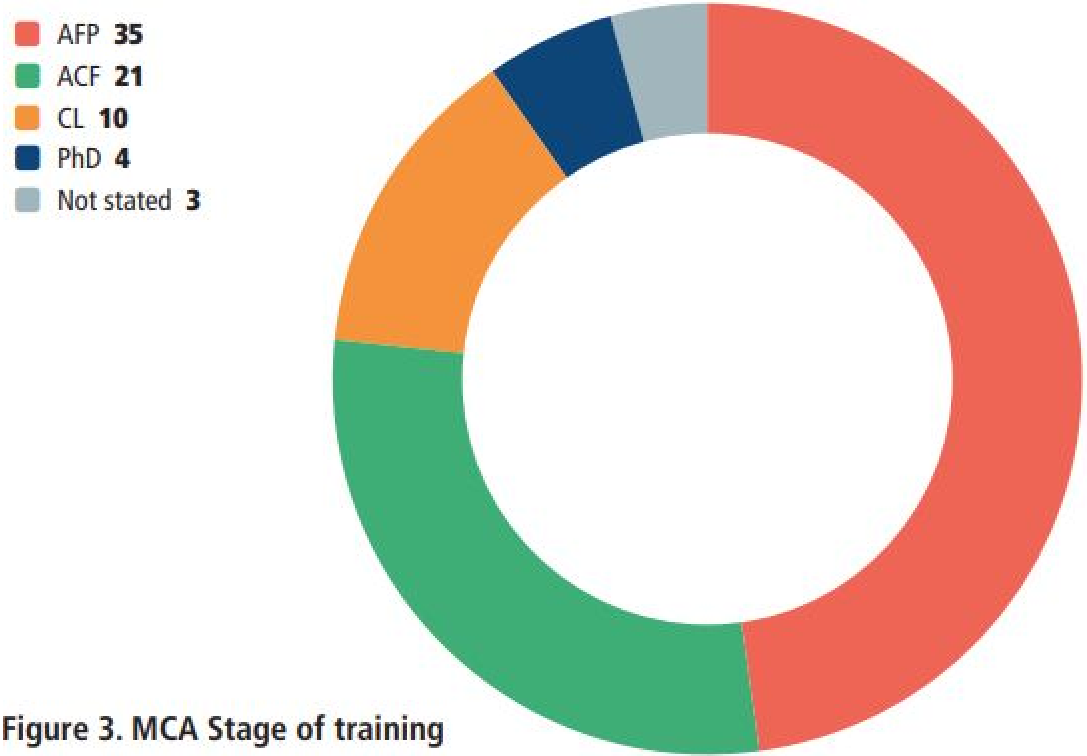

Figure 3. MCA Stage of training

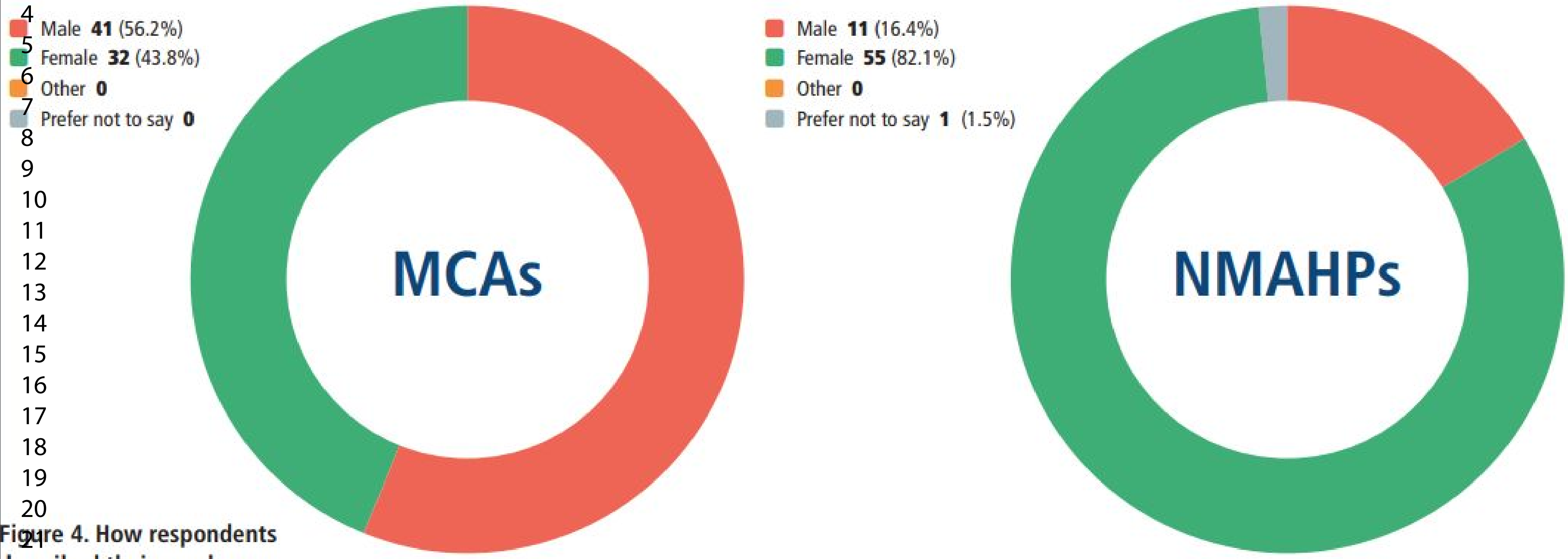

Figure 4. How respondents described their gender

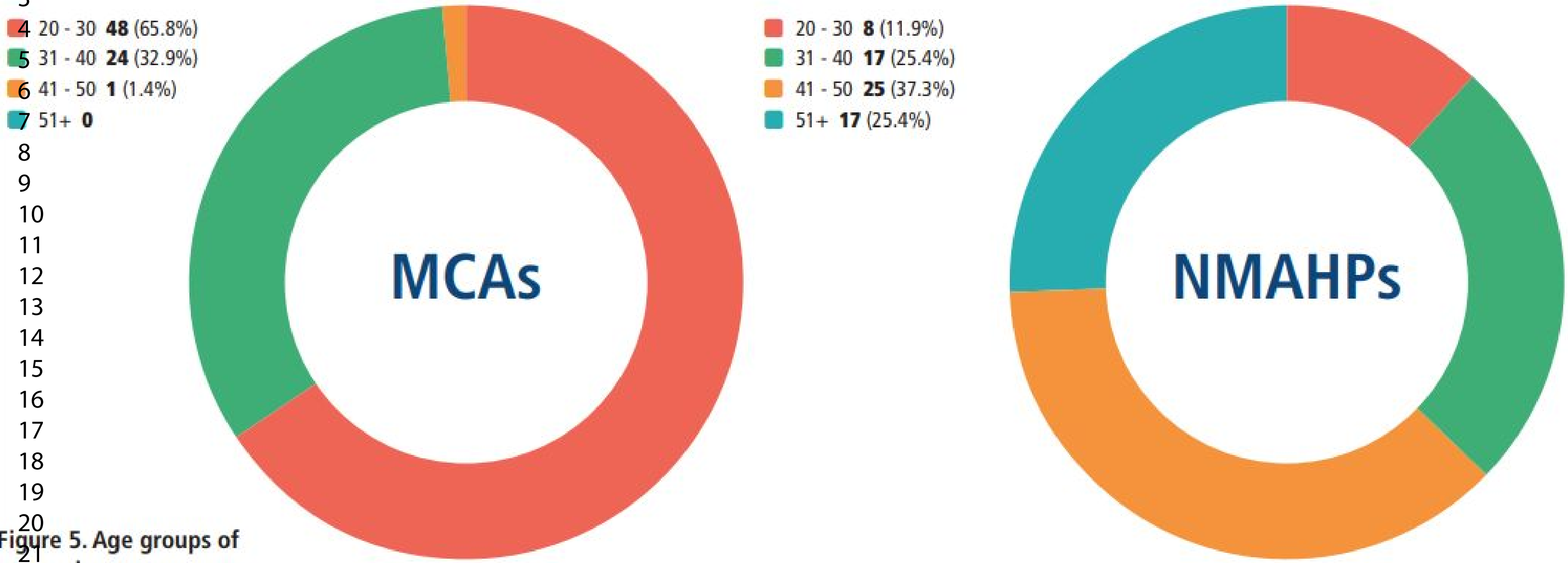

Figure 5. Age groups of respondents

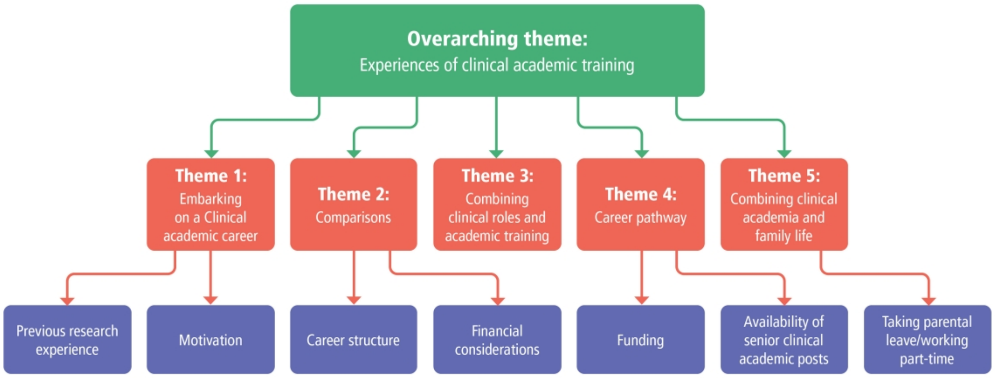

Figure 6. Coding tree.

275x106mm (300 x 300 DPI)

Standards for Reporting Qualitative Research (SRQR)

O'Brien B.C., Harris, I.B., Beckman, T.J., Reed, D.A., & Cook, D.A. (2014). Standards for reporting qualitative research: a synthesis of recommendations. *Academic Medicine, 89(9)*, 1245-1251.

No. Topic	Item	Page
**Title and abstract**		
S1 Title	Concise description of the nature and topic of the study identifying the study as qualitative or indicating the approach (e.g., ethnography, grounded theory) or data collection methods (e.g., interview, focus group) is recommended	1
S2 Abstract	Summary of key elements of the study using the abstract format of the intended publication; typically includes objective, methods, results, and conclusions	2
**Introduction**		
S3 Problem formulation	Description and significance of the problem/phenomenon studied; review of relevant theory and empirical work; problem statement	3-5
S4 Purpose or research question	Purpose of the study and specific objectives or questions	4-5
**Methods**		
S5 Qualitative approach and research paradigm	Qualitative approach (e.g., ethnography, grounded theory, case study, phenomenology, narrative research) and guiding theory if appropriate; identifying the research paradigm (e.g., positivist, constructivist/interpretivist) is also recommended	5
S6 Researcher characteristics and reflexivity	Researchers' characteristics that may influence the research, including personal attributes, qualifications/experience, relationship with participants, assumptions, or presuppositions; potential or actual interaction between researchers' characteristics and the research questions, approach, methods, results, or transferability	8
S7 Context	Setting/site and salient contextual factors; rationale[a]	5
S8 Sampling strategy	How and why research participants, documents, or events were selected; criteria for deciding when no further sampling was necessary (e.g., sampling saturation); rationale[a]	5-6, 8
S9 Ethical issues pertaining to human subjects	Documentation of approval by an appropriate ethics review board and participant consent, or explanation for lack thereof; other confidentiality and data security issues	8-9
S10 Data collection methods	Types of data collected; details of data collection procedures including (as appropriate) start and stop dates of data collection and analysis, iterative process, triangulation of sources/methods, and modification of procedures in response to evolving study findings; rationale[a]	6-8
S11 Data collection	Description of instruments (e.g., interview guides, questionnaires)	7-8

For peer review only

instruments and technologies	and devices (e.g., audio recorders) used for data collection; if/how the instrument(s) changed over the course of the study	
S12 Units of study	Number and relevant characteristics of participants, documents, or events included in the study; level of participation (could be reported in results)	6, 10
S13 Data processing	Methods for processing data prior to and during analysis, including transcription, data entry, data management and security, verification of data integrity, data coding, and anonymization/deidentification of excerpts	9
S14 Data analysis	Process by which inferences, themes, etc., were identified and developed, including researchers involved in data analysis; usually references a specific paradigm or approach; rationale[a]	10
S15 Techniques to enhance trustworthiness	Techniques to enhance trustworthiness and credibility of data analysis (e.g., member checking, audit trail, triangulation); rationale[a]	10
**Results/Findings**		
S16 Synthesis and interpretation	Main findings (e.g., interpretations, inferences, and themes); might include development of a theory or model, or integration with prior research or theory	10-21
S17 Links to empirical data	Evidence (e.g., quotes, field notes, text excerpts, photographs) to substantiate analytic findings	10-21
**Discussion**		
S18 Integration with prior work, implications, transferability, and contribution(s) to the field	Short summary of main findings; explanation of how findings and conclusions connect to, support, elaborate on, or challenge conclusions of earlier scholarship; discussion of scope of application/generalizability; identification of unique contribution(s) to scholarship in a discipline or field	21-24
S19 Limitations	Trustworthiness and limitations of findings	24-25
**Other**		
S20 Conflicts of interest	Potential sources of influence or perceived influence on study conduct and conclusions; how these were managed	26
S21 Funding	Sources of funding and other support; role of funders in data collection, interpretation, and reporting	26

[a]The rationale should briefly discuss the justification for choosing that theory, approach, method, or technique rather than other options available, the assumptions and limitations implicit in those choices, and how those choices influence study conclusions and transferability. As appropriate, the rationale for several items might be discussed together.

For peer review only

Clinical Academic Survey

Clinical academic survey

The East Midlands Clinical Academic Steering Group are hoping to demonstrate the impact of clinical academic training programmes so that future cohorts are able to benefit from the same opportunities that you have had.

With this in mind, we would be extremely grateful if you could take a few minutes to complete this questionnaire in Part 1 and also, if possible, to answer the additional questions in Part 2 (approx. 10 minutes). Part 3 is for people who are currently on a clinical academic training programme.

We appreciate that some work on this has already been undertaken by others, so if you have already taken part in a similar survey, we do not expect you to repeat the information.

Please be assured that all of your responses will be treated confidentially. Also, any quotes from the data that we may use when disseminating the results, will be anonymised.

All returned responses will be entered into a **prize draw** for a chance to **win a book of your choice up to a value of £50**. Please return the form by **20th April, 2018** and provide your name and email address here so that we can contact the prize winner.

For peer review only - http://bmjopen.bmj.com/site/about/guidelines.xhtml

Thank you very much in advance for your help. Good luck in the prize draw!

For peer review only

For peer review only - http://bmjopen.bmj.com/site/about/guidelines.xhtml

Part 1

Age group *Optional*

- ○ 20-30
- ○ 31-40
- ○ 41-50
- ○ 51+

How would you describe your gender?

- ○ Male
- ○ Female
- ○ Other
- ○ Prefer not to say

Current employment

- ☐ NHS
- ☐ University
- ☐ Both NHS and University
- ☐ Other employer

Please give the name(s) of your employer(s)

For peer review only - http://bmjopen.bmj.com/site/about/guidelines.xhtml

Please give your job title(s) and professional role (e.g assistant professor and nurse)

Are you employed:

- Full-time
- Part-time

At what level? (e.g. AFC Band or HEI scale)

For peer review only - http://bmjopen.bmj.com/site/about/guidelines.xhtml

Training programmes

Which of these clinical academic training programmes have you undertaken/are you undertaking? Please tick all that apply:

- ☐ Bronze fellowship
- ☐ Silver fellowship
- ☐ MARM
- ☐ NIHR funded MARM
- ☐ Masters in Clinical Research Studentship
- ☐ Other Masters programme
- ☐ Professional Doctorate
- ☐ NIHR funded PhD
- ☐ PhD not funded by NIHR
- ☐ Gold fellowship
- ☐ Other

Where did you undertake each training? (please list details for all courses that you have done) ✱ *Required*

How was your PhD funded?

If you selected Other, please specify: **Was it clinical academic training or another type of training?** Please provide details such as training provider; course completed/predicted completion date; qualification:

Which is the last training that you have done and when did it/will it finish? (month/year)

What were your reasons for stopping training at that point?

For current PhD students: When did you start your PhD? (month/year)

Is it NIHR funded?

- Yes
- No

If not, how is your PhD funded?

Where are you on the PhD journey?

For peer review only - http://bmjopen.bmj.com/site/about/guidelines.xhtml

- ☐ Year 1
- ☐ Year 2
- ☐ Year 3
- ☐ Year 4
- ☐ Year 5
- ☐ Year 6
- ☐ Thesis pending

What is your anticipated end date? (month/year)

For peer review only

For peer review only - http://bmjopen.bmj.com/site/about/guidelines.xhtml

Please give brief details below of what you have done as a consequence of your funding/training:

Have you changed your employment in any way? For example, new roles; grades etc?

Please describe ways in which your training has had an impact or changed your clinical practice:

Have you disseminated your research:

- ☐ Locally
- ☐ Nationally
- ☐ Both

Please provide details of how you have disseminated your research (e.g. publications, conference presentations/posters). If possible, please provide links or cut and paste details.

For peer review only - http://bmjopen.bmj.com/site/about/guidelines.xhtml

Have you had any successful grant applications or other collaborations?

- Yes
- No

If so, please provide details (e.g. the funding body, research project, grant reference (if any), and whether you were the lead or co-applicant)

Thank you for the answers you have provided so far. We would be grateful if you could also take a few minutes to complete some or all of the questions in part 2. Current clinical academics are also invited to complete part 3. Otherwise, please skip to the finish at the end of part 3 to submit your survey.

- Proceed to part 2
- Skip to part 3

For peer review only - http://bmjopen.bmj.com/site/about/guidelines.xhtml

Part 2

In this part of the questionnaire, we want to find out what the term ‘clinical academic’ means to you, and how the clinical academic training you have received has influenced your career.

The description ‘clinical academic’ is varied. We want to try to find a way to best explain what the role is. Please explain what you understand by the title ‘clinical academic’:

Do you consider yourself to be a clinical academic?

- Yes
- No

If so why? (for example, do you have funded research and clinical sessions?)

How has the clinical academic training you have received helped you in your career and/or clinical practice?

For peer review only - http://bmjopen.bmj.com/site/about/guidelines.xhtml

How did you overcome any challenges in achieving success and/or progressing to the next level in your clinical academic career?

What types of support did you find most helpful during your clinical academic training?

Have your goals changed during your training?

- Yes
- No

If so, please explain how and why your goals have changed:

For peer review only - http://bmjopen.bmj.com/site/about/guidelines.xhtml

Are there any resources that you found beneficial in developing your clinical academic role which you would be happy to share for the benefit of others?

These might include, for example, a job description or a report which was helpful in gaining the support of your managers for your training.

If you have any resources that you think might be useful, please send them as an attachment to the following email: diane.trusson@nottingham.ac.uk . Alternatively, please give link(s) to electronic resources here:

What advice would you give to people who are considering undertaking training and embarking on a clinical academic career?

Is there anything else that you want to add?

If you are currently on a clinical academic training programme (e.g. MRes/MARM/Bronze or Silver scholar/PhD/Post-doc), please complete the questions in Part 3. Otherwise, please press 'next' to finish on the final page.

For peer review only - http://bmjopen.bmj.com/site/about/guidelines.xhtml

Part 3. If you are currently on a clinical academic training programme e.g. MRes/MARM/Bronze or Silver scholar/PhD/Post-doc

Would you be interested in taking part in a focus group to discuss your experiences?

- Yes
- No

If so, please provide your contact details here:

We would also like to develop some case studies to demonstrate the impact that clinical academic training can have both in relation to your career and your clinical practice. Would you be willing to be interviewed about your experiences?

- Yes
- No

If so, please provide your contact details here:

For peer review only - http://bmjopen.bmj.com/site/about/guidelines.xhtml

Thank you for taking the time to complete this survey, your responses are very important to us.

For peer review only

For peer review only - http://bmjopen.bmj.com/site/about/guidelines.xhtml

Clinical Academic Survey for medics

Page 1: Clinical academic survey

Health Education England (HEE) PG Specialty School of Clinical Academic Training in the East Midlands

We are sending you this survey because we are interested in finding out more about your experiences of pursuing a clinical academic pathway. A similar survey has already been completed by nurses, midwives and allied health professionals in the East Midlands. The aim of this further study is to compare clinical academic experiences of different professions.

Completing the survey will take approximately 10 minutes and as a thank you for your time there is a chance to **win a £50 Amazon voucher**.

Please be assured that all of your responses will be treated confidentially. Also, any quotes from the data that we may use when disseminating the results, will be anonymised.

All returned responses will be entered into a **prize draw** for a chance to **win a £50 Amazon voucher!** Please return the form by midnight on **15th July, 2019** and provide your name and email address here so that we can contact the prize winner.

Thank you very much in advance for your help. Good luck in the prize draw!

For peer review only - http://bmjopen.bmj.com/site/about/guidelines.xhtml

Page 2: Part 1 - About you

Which age group are you in? *Optional*

- 20-30
- 31-40
- 41-50
- 51+

How would you describe your gender?

- Male
- Female
- Other
- Prefer not to say

How would you describe your ethnicity? (optional)

Current employment

- NHS
- University

Who are you employed by?

What is the name of the clinical practice where you are employed?

Which postgraduate specialty school are you a member of?

- [ ] Foundation School
- [ ] School of Anaesthesia
- [ ] School of Emergency Medicine
- [ ] Primary Care
- [ ] School of Medicine
- [ ] Obstetrics and Gynaecology
- [ ] Paediatrics
- [ ] Pathology
- [ ] Psychiatry
- [ ] Public Health
- [ ] Radiology
- [ ] Surgery

Please tell us the name of your clinical training programme

For peer review only - http://bmjopen.bmj.com/site/about/guidelines.xhtml

Page 3: Do you have a PhD/MD or are you studying for one?

If you are studying for a PhD, which University is your PhD affiliated with?

- ☐ University of Leicester
- ☐ University of Nottingham
- ☐ Other

Please give the name of University that your PhD is affiliated with

For current PhD/MD students: When did you start your PhD/MD? (month/year)

Who is funding your PhD?

Please give details of your PhD funding

Where are you on the PhD/MD journey?

- ☐ Year 1

For peer review only - http://bmjopen.bmj.com/site/about/guidelines.xhtml

- ☐ Year 2
- ☐ Year 3
- ☐ Year 4
- ☐ Year 5
- ☐ Year 6
- ☐ Submitted but not awarded

What is your anticipated submission date? (month/year)

For peer review only - http://bmjopen.bmj.com/site/about/guidelines.xhtml

Page 4

Please give brief details below of what you have done as part of your academic training:

Please describe ways in which your academic training has improved your skills as a clinician

Have you presented your research:

- ☐ Locally
- ☐ Nationally
- ☐ Internationally
- ☐ All the above

Please provide details of how you have disseminated your research (e.g. publications, conference presentations/posters). If possible, please provide links or cut and paste details.

Have you had any successful grant applications?

For peer review only - http://bmjopen.bmj.com/site/about/guidelines.xhtml

- Yes
- No

If so, please provide details (e.g. the funding body, research project, grant reference (if any), and whether you were the lead or co-applicant)

For peer review only

For peer review only - http://bmjopen.bmj.com/site/about/guidelines.xhtml

Page 5: Part 2. Becoming a clinical academic

In this part of the questionnaire, we want to find out what the term ‘clinical academic’ means to you, and how the clinical academic training you have received has influenced your career.

The description ‘clinical academic’ is varied. We want to try to find a way to best explain what the role is. Please explain what you understand by the title ‘clinical academic’:

Do you consider yourself to be a clinical academic?

- Yes
- No

If so why? (for example, do you have funded research and clinical sessions?)

How has your academic training helped you in your career? (if applicable)

If applicable, how did you overcome any challenges in achieving success and/or progressing to the next level in your clinical academic career?

What types of support did you find most helpful during your clinical academic training?

Do you anticipate any challenges in pursuing a clinical academic career? For example if you want to take breaks for parental leave or work part time in the future?

Have your goals changed during your training?

- Yes
- No

If so, please explain how and why your goals have changed:

For peer review only - http://bmjopen.bmj.com/site/about/guidelines.xhtml

For peer review only

For peer review only - http://bmjopen.bmj.com/site/about/guidelines.xhtml

Page 6

Are there any resources that you found beneficial in developing your clinical academic role which you would be happy to share for the benefit of others?

These might include, for example, websites where you have found useful advice and information.

If you have any resources that you think might be useful, please send them as an attachment to the following email: diane.trusson@nottingham.ac.uk . Alternatively, please give link(s) to electronic resources here:

What advice would you give to people who are considering a clinical academic career?

Is there anything else that you want to add?

For peer review only - http://bmjopen.bmj.com/site/about/guidelines.xhtml

Page 7: Part 3. Would you be willing to talk about your experiences?

Would you be interested in taking part in a focus group to discuss your experiences?

- Yes
- No

If so, please provide your contact details here:

We would also like to develop some case studies to demonstrate the impact that undertaking a clinical academic pathway can have both in relation to your career and your clinical practice. Would you be willing to be interviewed about your experiences?

- Yes
- No

If so, please provide your contact details here:

For peer review only - http://bmjopen.bmj.com/site/about/guidelines.xhtml

Page 8: Thank you for taking the time to complete this survey, your responses are very important to us.

Key for selection options

6.a - Who are you employed by?

University of Leicester
University of Nottingham
University Hospitals of Leicester
Nottingham University Hospitals
Partnership Trust
Other

8.a - Who is funding your PhD?

NIHR
Wellcome Trust
Medical Research Council (MRC)
Other

For peer review only
